# Accurate prediction of protein relative solvent accessibility using a balanced model

**DOI:** 10.1186/s13040-016-0121-5

**Published:** 2017-01-24

**Authors:** Wei Wu, Zhiheng Wang, Peisheng Cong, Tonghua Li

**Affiliations:** Department of Chemistry, Tongji University, Shanghai, China

**Keywords:** Balanceable model, Prediction, Profile, Relative solvent accessibility

## Abstract

**Background:**

Protein relative solvent accessibility provides insight into understanding protein structure and function. Prediction of protein relative solvent accessibility is often the first stage of predicting other protein properties. Recent predictors of relative solvent accessibility discriminate against exposed regions as compared with buried regions, resulting in higher prediction accuracy associated with buried regions relative to exposed regions.

**Methods:**

Here, we propose a more accurate and balanced predictor of protein relative solvent accessibility. First, we collected known proteins in three subsets according to sequence length and constructed a balanced dataset after reducing redundancy within each subset. Next, we measured the performance associated with different variables and variable combinations to determine the best variable combination. Finally, a predictor called BMRSA was constructed for modelling and prediction, which used the balanced set as the training set, the position- specific scoring matrix, predicted secondary structure, buried-exposed profile, and length of a query sequence as variables, and the conditional random field as the machine-learning method.

**Results:**

BMRSA performance on test sets confirmed that our approach improved prediction accuracy relative to state-of-the-art approaches and was balanced in its comparison of buried and exposed regions. Our method is valuable when higher levels of accuracy in predicting exposed-residue states are required. The BMRSA is available at: http://cheminfo.tongji.edu.cn:8080/BMRSA/.

**Electronic supplementary material:**

The online version of this article (doi:10.1186/s13040-016-0121-5) contains supplementary material, which is available to authorized users.

## Background

Since the concept of protein solvent accessibility was introduced in protein structures [1], solvent accessibility has been considered as an important measure of spatial arrangement during the process of protein folding. Given that the solvent accessibility of an amino acid in a protein defines its surrounding solvent environment and hydration properties, this characteristic has been widely used to analyze protein structure and function. Prediction of relative solvent accessibility (RSA) is often the first predictive stage of determining protein structure and function. Predicted RSA assists in predicting protein secondary structure [2–4], domain boundary [5], disorder [6–8] and hot spot [9], as well as protein-protein interaction prediction [10] and fold recognition [11]. Recently a number of new methods were developed to predict RSA [12–15].

Traditionally, RSA prediction is treated as a multi-class classification problem. However, it is often transformed into a binary classification according to a defined threshold of RSA values. Threshold definitions vary, however, in most cases for comparing with other methods the threshold is defined as 25% of RSA value, resulting in a residue being classified as buried (defined as the RSA value is less than 25%) or exposed.

Machine learning-based methods are the most successful methods for RSA prediction from amino acid sequences. However, Network–based regression methods [16], fuzzy k-nearest neighbor [17], support vector machine [18] and random forest [19] etc. approaches have been explored for RSA prediction.

With continual advances in technology, RSA prediction accuracy has increased over 80%. Recently, outstanding RSA predictors, capable of providing large-scale RSA prediction, have been implemented and perform better than other approaches [20–22]. However, we find that these methods discriminate against potential exposed state residues. Prediction accuracy of buried state residues is often higher relative to the exposed state. This is unfortunate, when given that properties associated with solvent exposed regions are considered more important than buried regions. For example, analysis of protein-protein interaction hot spots indicates that they are frequently located on the protein surface. One potential problem associated with this defect in existing prediction methods may be unbalanced training sets. Prediction requires large non-redundant training sets, which are frequently obtained using CD-HIT [23] or PISCES [24]. However, these tools reserve the longest sequences to represent a clustered group, while shorter sequences are removed from the training sets. Differing from other one-dimensional structural characteristics, residue RSA value is impacted not only by its own orientation and that of its neighbors, but also by other residues located elsewhere in the protein structure. Due to spatial contacts, a residue within a longer sequence is more easily buried relative to one found in a shorter sequence. Thus, a training set that lacks shorter sequences that may represent exposed protein regions is unlikely to accurately predict exposed sites.

Although the position-specific scoring matrix (PSSM) and predicted secondary structure are considered appropriate variables for RSA prediction, it is believed that more effective variables should be explored to improve RSA prediction accuracy.

Here, we present a novel balanced model for RSA prediction from the amino acid sequence. We constructed a balanced training set according to the lengths of known sequences and proposed a new ‘buried-exposed profile’ variable, which is obtained via sequence-based structure similarity. Using the balanced training set and the optimized variable combination, we built a balanced model for RSA prediction. Results indicate that our method is a more accurate predictor of and a more balanced model for RSA prediction relative to state-of-the-art approaches.

## Materials and methods

The accessible surface area of a residue in a protein chain is firstly calculated by DSSP [25] and then divided by the maximum solvent accessibility according to Chothia’s work [26] which uses Gly-X-Gly extended tripeptides, so that the RSA value of a residue could be obtained. In units Å^2^, these are 210 (Phe), 175 (Ile), 170 (Leu), 155 (Val), 145 (Pro), 115 (Ala), 75 (Gly), 185 (Met), 135 (Cys), 255 (Trp), 230 (Tyr), 140 (Thr), 115 (Ser), 180 (Gln), 160 (Asn), 190 (Glu), 150 (Asp), 195 (His), 200 (Lys), and 225 (Arg).

### Data sets

A template library was constructed, wherein the sequences and buried-exposed states were obtained from the Protein Data Bank (PDB) [25]. Sequences up to December 31, 2013 (89,135 entries), longer than 40 amino acids were collected. Sequences from the template library were obtained using PISCES [24] with a sequence identity threshold of 99%, resolution <2.5 Å, and an R-value <0.2. The RSA value was calculated by using DSSP [27]. This returned 28,155 entries with sequences and the buried-exposed elements were saved as a single file using FASTA format.

A training set should contain abundant and diverse buried and exposed paradigms. After collecting the template library, the sequences were divided into three subsets according to sequence length, with length thresholds set to 40, 150 and 300 residues to assure we had enough buried and exposed paradigms. The similarity between pairwise sequences limited to 25% sequence identity using PISCES [24] for three subsets. There are 3274, 2792, and 2230 sequences remaining in the short-, medium-, and long-length subset, respectively. We merged the remaining entries from three subsets into a training set containing 8296 protein chains (DB8296). The PDB IDs of DB8296 are listed in the Additional file 1: S1. In DB8296, there are 1,798,501 residues, with 877,347 residues (48.8%) residing in buried regions. Due to the redundancy reduction was undertaken for each subset, there remained some short homologous sequences in DB8296. However, these sequences contain rich exposed paradigms that must be contained in the training set (see next section).

The selection process of test set (DB101) was similar to that used for DB8296. Sequences in the PDB [26] deposited between January 1, 2014 and June 2, 2015, and having lengths ≥40 amino acids were selected (11,900 entries). Similarity of pairwise sequences was cutoff at 25% sequence identity, with resolution <2.5 Å and an *R*-value <0.2. Remaining entries were combined with the template library and redundancy was further reduced using a 25% sequence-identity threshold. After further analysis, a remaining sequence having <25% sequence identity with each sequence in the template library was selected to construct the independent test set, resulting in 101 non-redundant protein chains. Thus the DB101 is independent with the training set and the template library. The PDB IDs of DB101 are listed in the Additional file 1: S2.

### Balanced training set

**Fig. 1:**
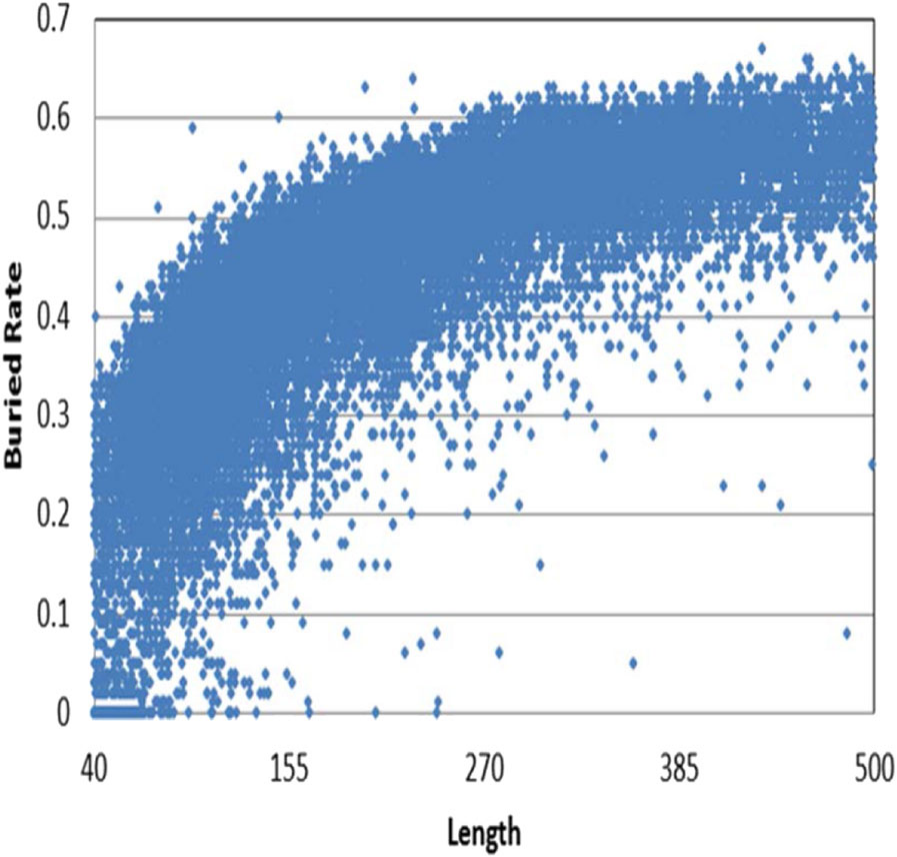
The relationship between the buried ratios and the sequence lengths

**Fig. 2:**
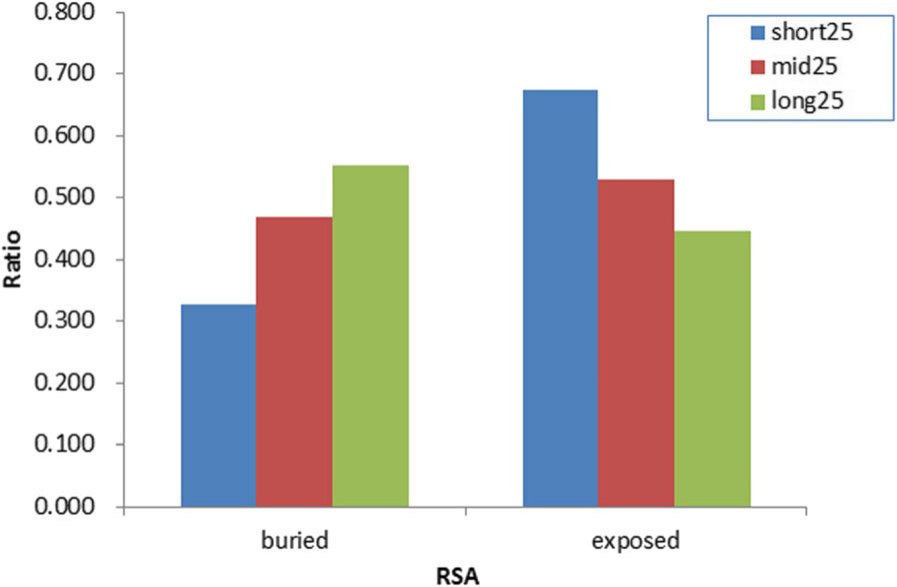
The ratios of buried and exposed residues in short-, medium- and long-length subsets

Our observations indicated a relationship between the ratios of buried residues present within sequences of length (40–500 residues, Fig. 1) and increases in the average value of ratios of buried residues and sequence length. The percentage of buried residues is <40% when the length is shorter than 150 residues and >50% when the length is longer than 300 residues. Therefore we divided all sequences in the template library into three subsets according to length thresholds (150 and 300 residues). Analysis of the number of buried and exposed residues in three subsets revealed that the ratios of buried to exposed residues differ (Fig. 2). In the short-length subset, the number of exposed residues is larger than that observed for buried residues. However, in the long-length subset, the number of exposed residues is smaller than that observed in for buried residues. This result may explain why existent predictors discriminate against exposed states. In these predictors, the training sequences were constructed by using reducing redundancy. However, the methods reserved the longest sequences and removed all shorter homologous sequences. Thus, the results from using those training sets predicted larger numbers of buried residues relative to exposed residues. In comparison with other structural characteristics, such as secondary structure, RSA defines both local and global structure. The RSA value of a residue is affected by its own structure, that of its neighbors, and those of distant residues. Due to spatial contacts, a similar residue in a longer sequence is more likely to be buried following protein folding than that found in a shorter sequence.

**Fig. 3:**
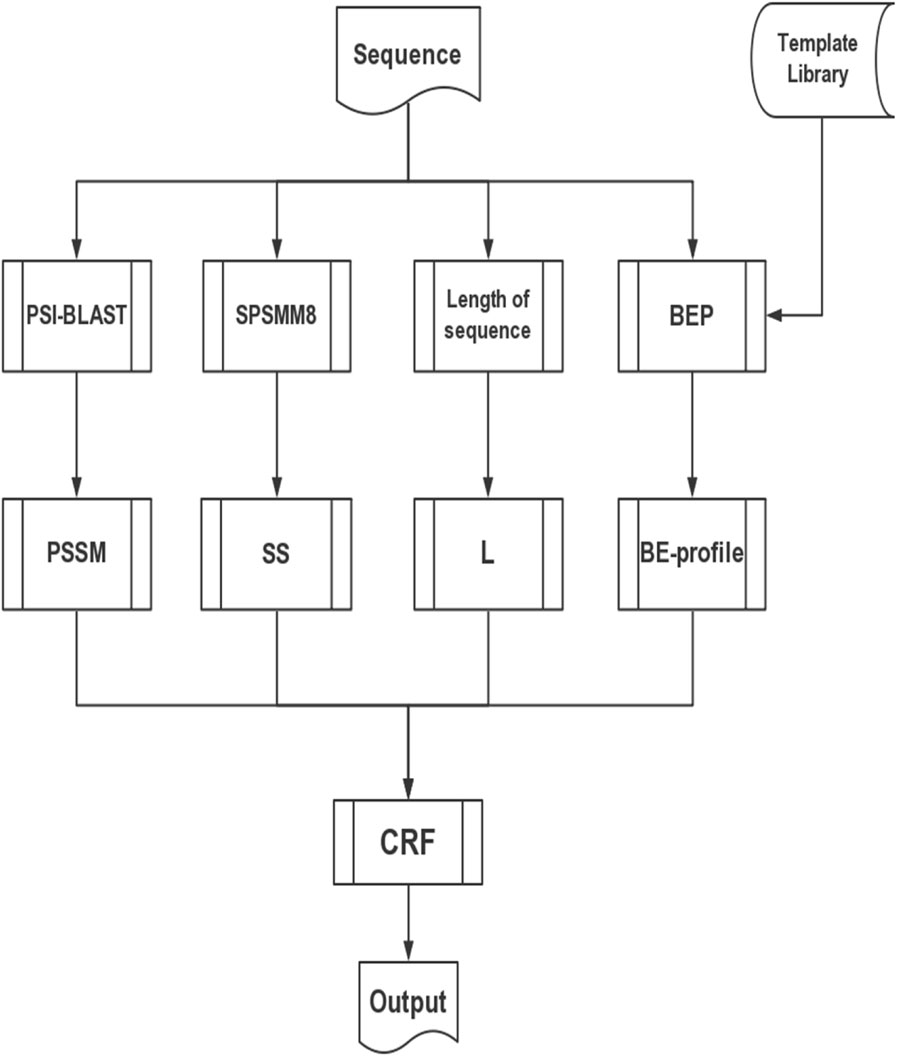
The flowchart of BMRSA

For example, in our training set there is a short chain (PDB ID: 1A6S-A, 87 residues), in which a fragment has high sequence identity (Score = 32.7 bits (73), Expect = 0.023, Method: Composition-based stats. Identities = 22/53 (41%), Positives = 29/53 (54%), Gaps = 3/53 (5%)) with a fragment in a long chain (PDB ID: 2ZZV-A, 361 residues).
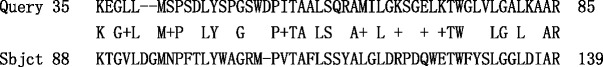


In these fragments here are 22 matched residues. However, their buried-exposed states are quite different.
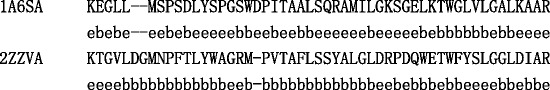


For 1A6S-A the number of the buried residues is 9 and the number of the exposed residues is 20. On the contrary, for 2ZZV-A, the number of the buried residues is 20 and the number of the exposed residues is 9. Therefore we think the short chain and the long chain should all be reserved in the training set for buried and exposed paradigms.

After the redundancy reduction (described above) we have attempted various methods at constructing more appropriate training sets. For example, when we used the long-length set as a training set, the buried-residue prediction accuracy was much higher relative to the exposed-residue prediction accuracy, especially for short-length test sequences. When we used the short-length subset as a training set, the exposed-residue prediction accuracy was >90% for short-length test sequences, however, the buried-residue prediction accuracy was <60%. For the long-length test set, the exposed-residue accuracy was >85%, however, the buried-residue prediction accuracy was very low, indicating a discrimination against buried-residue states. We recognized that a balanced training set was necessary for a balanced model of RSA prediction. We merged short-length, medium-length, and long-length sequences as our balanced training set, resulting in a number of buried residues approximately equal to the number of exposed residues.

### The buried-exposed profile

The program of accurate and balanced RSA prediction must explore valuable variables. We have assessed five types of variables and their combinations. Here, we propose a new variable referred to a buried-exposed profile (BE-profile). The BE-profile comprises structural evolution information obtained via sequence-based structure similarity.

A query sequence was aligned against the template library using PSI-BLAST [28] with an e-value of 1e-5, the number of iterations set to three, and the remaining parameters set as default values. Homologous sequences selected according to e-value were obtained in ascending order, with the top S (the default is ten) of these sorted sequences considered to contain rich homologous information and reserved (if the number of the selected sequences is less than S, all selected sequences will be retained).

If an amino acid in the query has been matched with residues from the homologous sequences, a BE-profile vector with two elements of the amino acid can be calculated in the form of probabilities of the buried and the exposed states. The BE-profile is defined as:

1 $$ \mathrm{BE}-\mathrm{profile}\ \left(p,t\right)={\displaystyle {\sum}_S{A}_S}\left(p,t\right)/{\displaystyle {\sum}_t{\displaystyle {\sum}_S{A}_S}}\left(p,t\right) $$

where p represents the position of the amino acid in the query sequence and t represents either the buried or exposed state. The variable s represents the number of matched sequences. A_S_(p,t) represents the state of an amino acid in s homologous sequence that matched with the amino acid in the query sequence constitutes a binary value. When the state is t, A_S_(p,t) is 1, otherwise it is 0. In the denominator, the summation is carried out for these two states.

The BE-profile constitutes a template-based variable that is obtained via sequence-based structure similarity and describes the probabilities of the buried and exposed states of a residue in an evolutionary procedure. The variable also contains conserved and evolutionary information and avoids conflicts between different buried and exposed states for the same residue in different matched sequences. A BE-profile is a dynamic statistical analysis of the properties of a residue. Thus, two identical residues in two different sequences, even within the same sequence, may have different BE-profile values.

### Performance evaluation

To measure the performance of RSA prediction with binary classification, the buried state is defined as positive and the exposed state as negative. Thus, sensitivity (defined as TP/(TP + FP)) can be considered as buried accuracy, and specificity (TN/(TN + FN)) as exposed accuracy, where TP represents the number of predicted true buried residues, FP represents the number of predicted false buried residues, TN represents the number of predicted true exposed residues, and FN represents the number of predicted false exposed residues. The total accuracy and MCC (Matthew’s correlation coefficient) are calculated as follows:

2 $$ \mathrm{Accuracy}=\frac{TP+TN}{TP+FP+TN+FN} $$

3 $$ \mathrm{M}\mathrm{C}\mathrm{C}=\frac{TPTN- FPFN}{\sqrt{\left(TN+FN\right)\left(TP+FN\right)\left(TN+FP\right)\left(TP+FP\right)}} $$

### The balanced model

Our balanced model, BMRSA, is based on the balanced training set and the optimized variables. The flowchart is shown in Fig. 3.

A query sequence is sent to four modules for RSA modelling and prediction. The PSI-BLAST module generates a PSSM vector with 20 elements for each residue in the query. The PSI-BLAST [28] parameters are set as defaults. The SPSSM8 module predicts eight-state secondary structure for a residue in the query. It is a one-element variable. The length of the sequence module measures the length of the query and generates an alphabet variable (S, M, L). The BE-profile module produces a BE-profile vector with two elements of a residue in the query. There are 24 variable elements in total.

When the queries are training sequences, a conditional random field (CRF) [29, 30] modelling routine is carried out to construct a model. When the queries consist of testing sequences, we use a prediction routine to predict the RSA based on the obtained model. CRFs are powerful probabilistic frameworks used to label and segment sequential data. CRFs are computationally fast and do not need sliding windows. In our approach, CRF++0.51 is used.

The keys to our balanced model are the balanced training set and the optimized variable combination, wherein the BE-profile and the length variable are important.

## Results and discussion

### Variable validation

In order to improve the performance of our predictor, we validated five types of variables using DB8296 as the training set and an independent test set (DB101).

A PSSM variable is a vector having 20 elements obtained via alignment using PSI-BLAST [25] (with all parameters set to default) against a non-redundant database. In agreement with previous studies, PSSM is an effective variable (Table 1), achieving 77.9% accuracy. The accuracy of exposed-residue prediction is 83.6%, higher than the 72.9% obtained for buried-residue prediction. This demonstrates that our training set has altered previous discrimination in favor of the exposed-residue state.

**Table 1:** Performance of different variables and their combinations

Variables	Sensitivity	Specificity	Accuracy	MCC
PSSM	0.729	0.836	0.779	0.565
PSSM + A-score	0.777	0.801	0.788	0.576
PSSM + SS	0.741	0.833	0.784	0.574
PSSM + ShapeString	0.782	0.806	0.793	0.586
PSSM + BE-profile	0.835	0.864	0.849	0.698
PSSM + BE-profile + SS	0.834	0.865	0.848	0.697
PSSM + BE-profile + ShapeString	0.836	0.863	0.849	0.698
PSSM + BE-profile + SS + L	0.859	0.867	0.863	0.723

Sequence metrics (reference A-score) [31] include five elements for each amino acid. A-score reflects basic residue properties and has been used to predict protein structure, including disordered regions [32]. Here, A-score was used as a variable for RSA prediction. Prediction accuracy improved slightly by using a combination of PSSM and A-score variables.

A predicted secondary structure (SS) variable is widely used in RSA prediction. We use SPSSM8 [33] to predict eight-state secondary structure from a sequence and use it in combination with PSSM as variables. The results indicate that the secondary structure variable contributed minimally to prediction accuracy, however, in more variable combinations, eight-state secondary structure still contributes.

Similar to secondary structure, a residue-shape string is also a representation of protein one-dimensional structure. The shape-string variable improves the accuracy of protein turn prediction [34]. We use a shape string predictor [35] to predict eight-state shape string of residues in the query. The shape-string variable combined with PSSM continuously improved prediction accuracy.

The BE-profile variable proposed in this study significantly improved prediction accuracy when coupled with PSSM as a variable. In two-variable combinations, the highest accuracy (84.9%) is achieved. The BE-profile is a distinctive variable when there exist sequence similarities between query sequences and the template library.

In three-variable combinations, secondary structure and the shape-string variable were combined with PSSM and BE-profile. The results indicate that the prediction accuracy was similar to previous methods.

A length variable was frequently used for RSA prediction. We also used it to assess whether CRF would utilize the short- or long-length training samples according to the length of a query sequence. As expected, the prediction accuracy achieved 86.3% and an MCC of 0.725 when the four-variable combination is used. Additionally, the sensitivity was 85.9% and the specificity was 86.7%. Thus, after optimization, PSSM, BE-profile, secondary structure, and length are determined as the optimal variables for our approach and constitute an accurate and balanced method for RSA prediction.

### Performance on DB101 and CASP11 targets

In order to compare our approach with other methods, we selected three RSA predictors considered to perform better than previous approaches and currently publically available. Table 2 shows the performances of different methods on DB101.

**Table 2:** Comparison of RSA predictors on DB101

Method	Sensitivity	Specificity	|Se-Sp|	Accuracy	MCC
NetSurfP	0.818	0.772	0.046	0.796	0.59
PaleAle 4.0	0.875	0.743	0.132	0.813	0.626
ACCpro5	0.877	0.804	0.073	0.843	0.684
RaptorX	0.754 (<0.1)	0.841 (>0.4)	0.087	0.807	0.595
BMRSA	0.859	0.867	0.008	0.863	0.725

NetSurfP [36] uses artificial neural networks to predict real and relative solvent accessibility of an amino acid. The training set used was relatively small (1764 entries) and old (sequences deposited before July 2007). NetSurfP RSA prediction accuracy using DB101 was 79.6%. Although the buried-residue prediction accuracy was higher than the exposed-residue prediction accuracy, the gap was 4.6% and constitutes an acceptable difference.

PaleAle is a series of 4-class (4, 25, and 50% thresholds) RSA predictors and PaleAle 4.0 [21] is a new version capable of using increasing training set sizes that result from the continued growth of the PDB. The training set used (7522 entries) was larger than previous sets [37], and the prediction accuracy using DB101 was 81.3%. However, the imbalance between buried- and exposed-residue prediction was >13%. Given that Porter 4.0 (secondary structure prediction) and PaleAle 4.0 shared the same training set, it was likely that differences between secondary structure and RSA prediction were ignored. The secondary structure associated with a residue in a protein is a local structural characteristic and is seldom affected by other long distance residues. However, the RSA of a residue is sometimes altered by other residues, even those separated by long distances. Therefore, Porter 4.0 is an unbalance model for RSA prediction.

ACCpro5 [22] is a new version of a series web server [38]. Similar to PaleAle, ACCpro also used the same training set for secondary structure prediction, however, it directly replaced previous predictions with known buried- and exposed-residue states in regions where similar sequences could be found in the PDB. Thus, the abundant exposed-residue information included in the known short-length sequences was utilized. The prediction accuracy of ACCpro5 was 84.3% and the specificity was 80.4%, which was higher than previously tested methods, but with a >7% gap.

RaptorX [13] is a new version of ACCpro and employed a powerful in-house deep learning model DeepCNF (Deep Convolutional Neural Fields) to improve the performance of RSA prediction. However, RaptorX is a three-state RSA predictor (buried (B) with RSA from 0 to 10%, intermediate (I) with RSA from 10 to 40% and exposed (E) with RSA from 40 to 100%), and could not directly be compared with our approach. In general, a two-class prediction is easier and outstanding than a three-state prediction. So we only take two classes performances of RaptorX for reference. The results are showed in Table 2. An interesting thing is it is also unbalance, but its specificity is higher than its sensitivity, which is contrary to other approaches. It reveals that we may have another way to improve unbalance between buried and exposed. To regulate two boundaries in 3-state RSA regions, we may construct a balance model for both buried and exposed. However, the performance of a 3-state predictor must be improved. In this example, the intermediate set has 8634 residues and is about 30% of all residues. But only 4534 residues were correctly predicted (the rate is 52.5%) by RaptorX. If it accounted into the performance, the measurements of RaptorX would be much worse than expectation.

Comparison with these methods mentioned above we use a larger training set (8296 entries), in which the short-length sequences that contained more exposed residues were added for balance. Similar with ACCpro5 our BMRSA also uses the known buried and exposed information from PDB, but using a different scheme. We construct BE-profile and considered it as a variable. BMRSA performs more accurate and more balance between the buried and the exposed states. The accuracy achieves 86.3, and 85.9 and 86.7% for buried and exposed respectively. The gap is only 0.8%. It demonstrates our approach significantly improves RSA prediction.

To further verify the performance of our approach, we assessed BMRSA on CASP11 (11^th^ Critical Assessment of Techniques for Protein Structure Prediction) targets and compared the results with other approaches. The CASP11 set (82 proteins) was downloaded from the official website. The most sequences of CASP11 targets have lower than 30% sequence identities with what have been stored in the PDB before 2014. Our purpose is to use the known structural information as much as possible to predict RSA of these targets. In CASP11 the buried ratio is 49.86%.

Table 3 shows the performances of four methods on CASP11. Due to CASP11 is a strict test set the accuracies of five methods all come down. Theoretically, RSA prediction exploits sequence profile and/or template information [13]. NetSurfP and PaleAle are sequence-based methods. Their predictive accuracies for CASP11 come down slightly (~2%) comparing with for DB101. ACCpro5 (also RaptorX) and our approach are sequence-based and template-based methods. So, they are usually excellent comparing with a sequence-based one when there are sequence homologies between a query and the templates (for example, DB101). However, if a query has a few or no sequence similarity with the templates (i.e. CASP11) the performance of a template-used approach would be affected greatly. Here, the accuracies of the template-based approaches come down about 6–7% and approximate to a sequence-based approach (PaleAle 4.0). It is worth pointing out that when a RSA predictor, for example a sequence-based model (PaleAle 4.0) or a template-based model (ACCpro5), used an unbalance non-redundant training set obtained by using CD-Hit or PISCES tool, its performance should be unbalance.

**Table 3:** Performance of RSA predictors using CASP11 dataset

Method	Sensitivity	Specificity	|Se-Sp|	Accuracy	MCC
NetSurfP	0.789	0.762	0.027	0.777	0.554
PaleAle 4.0	0.851	0.730	0.121	0.791	0.582
ACCpro5	0.816	0.746	0.070	0.781	0.563
RaptorX	0.726 (<0.1)	0.823 (>0,4)	0.097	0.789	0.545
BMRSA	0.781	0.806	0.025	0.793	0.587

PaleAle 4.0 achieves the best buried accuracy of 85.1%. However the gap is still over 12%. Our approach achieves the best accuracy of 79.3% and the best exposed accuracy 80.6% (except RaptorX). The gap is 2.5%. It again confirms BMRSA is an outstanding RSA predictor especially when the exposed accuracy is considered more important. According to defined B/I/E states by RaptorX the residue ratio of CASP11 is 0.33:0.31:0.36. The Q3 was 0.663 reported by RaptorX [13]. Due to the intermediate did not be included in our measurements, the sensitivity and the specificity of RaptorX listed in Table 2 just are references.

### Special examples

**Fig. 4:**
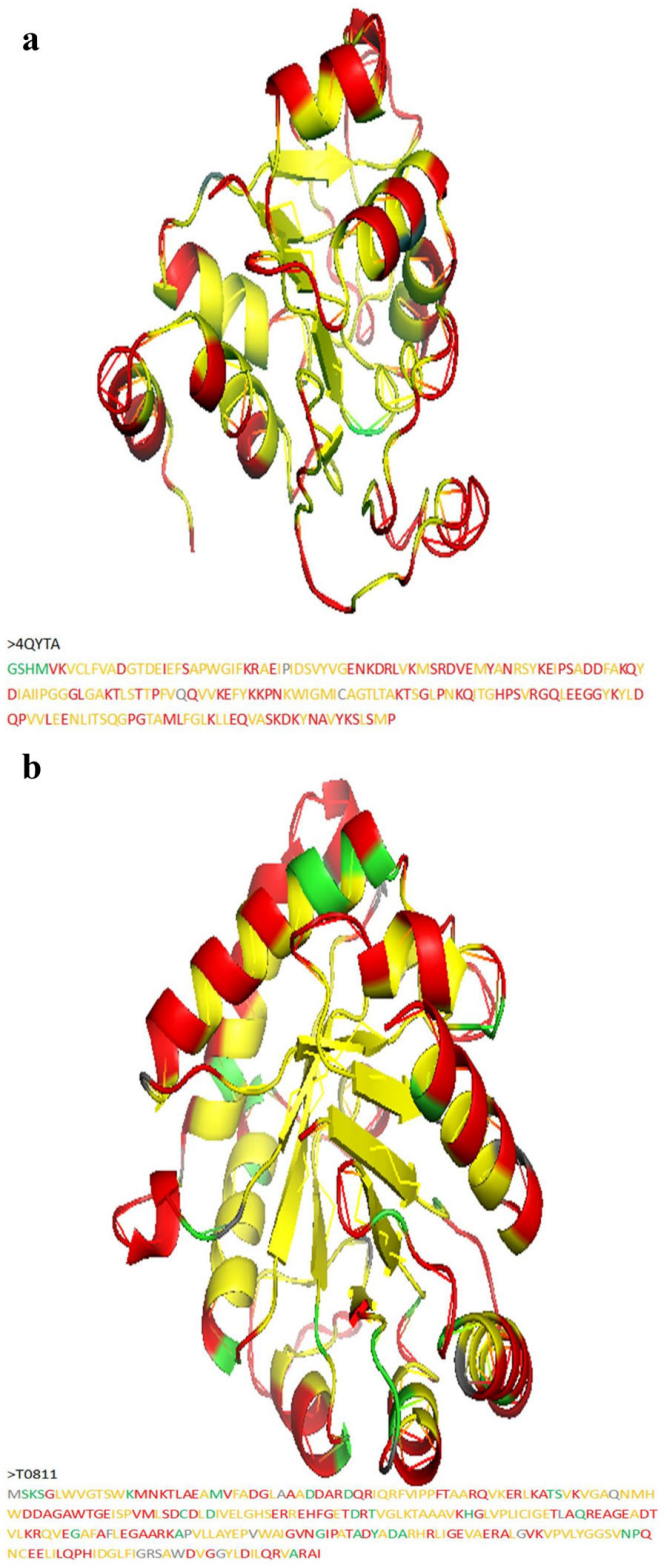
Two examples of RSA prediction. **a** 4QYT-(**a)**; (**b**) T0811. The predicted true buried residue is indicated by *yellow* and the predicted false buried residue by *gray*. The predicted true exposed residue is indicated by *red* and the predicted false buried residue by *green*

Two special examples are used to illustrate BMRSA performance. The three-dimensional structure of a *Schizosaccharomyces pombe* protein (PDB ID: 4QYT-A; 195 residues) selected from DB101 is shown in Fig. 4a. The BMRSA overall prediction accuracy was 98% with a sensitivity of 99% and a specificity of 98%. A CASP11 target (T0811, 255 residues) is a triosephosphate isomerase and is shown in Fig. 4b. The BMRSA overall prediction accuracy was 86% with a sensitivity of 84% and a specificity of 88%.

The results of two special examples provide insight into the varieties of solvent accessibility. The ratio of buried residues to exposed residues is 0.558 and 0.542 for 4QYT-A and T0811, respectively. The number of exposed residues is less than the number of buried residues. Aside from sequences located at the protein surface, exposed and buried residues often appear alternately in protein sequences. When a residue resides in a coil structure its status as either exposed or buried depends upon the orientation of the coil relative to the overall protein tertiary structure. When a helical structure is located on the surface of a protein, half of its residues orient outward and are solvent exposed, while the other half orient toward the inside of the protein and are considered buried. In these two examples, the residues of the β-sheet structures are all considered buried.

## Conclusion

In this study, we described a balanced model of RSA prediction based on a balanced training set and an optimized variable combination. Our approach is simple but performs better than state-of-the-art approaches. Specifically, BMRSA is capable of predicting exposed-residue states more accurately relative to buried-residue states. We believe that the balanced model will be widely used when higher levels of accuracy for predicting exposed-residue states are required and where interactions between a protein and its surroundings are of interest.

## Additional file

Additional file 1:
**S1.** The PDB IDs of DB8296. **S2.** The PDB IDs of DB101. (DOCX 101 kb)
