# Supplementary material for: Accurate prediction of protein relative solvent accessibility using a balanced model
Source: BioData Min. 2017 Jan 24;10:1. doi: 10.1186/s13040-016-0121-5 (PMC5259893; doi:10.1186/s13040-016-0121-5)
Supplement: Additional file 1: — S1. The PDB IDs of DB8296. S2. The PDB IDs of DB101. (DOCX 101 kb) [file 13040_2016_121_MOESM1_ESM.docx]

Additional file 1

**S1 The PDB IDs of DB8296**

| 12ASA | 16VPA | 1A3CA | 1A4IA | 1A6MA |
| --- | --- | --- | --- | --- |
| 1A6SA | 1A79A | 1A8DA | 1A8LA | 1A8RA |
| 1A8VA | 1A9VA | 1A9XB | 1ABAA | 1ABVA |
| 1ABZA | 1AC0A | 1AC5A | 1ACIA | 1ADNA |
| 1AE9A | 1AFOA | 1AGGA | 1AHLA | 1AHOA |
| 1AIWA | 1AJYA | 1AK6A | 1AL3A | 1AM2A |
| 1AM7A | 1AMMA | 1AMXA | 1AOAA | 1AOCA |
| 1AOYA | 1AQ5A | 1AQSA | 1ARBA | 1ASHA |
| 1ATGA | 1ATZA | 1AUUA | 1AVQA | 1AX8A |
| 1AYJA | 1AYOA | 1B0NB | 1B1CA | 1B25A |
| 1B2PA | 1B5EA | 1B67A | 1B68A | 1B6AA |
| 1B8DA | 1B8WA | 1B93A | 1BAGA | 1BAMA |
| 1BB8A | 1BBGA | 1BBNA | 1BBYA | 1BCOA |
| 1BDSA | 1BEAA | 1BG6A | 1BG8A | 1BGEA |
| 1BGFA | 1BGVA | 1BHEA | 1BHUA | 1BI6H |
| 1BIAA | 1BIFA | 1BINA | 1BKRA | 1BM8A |
| 1BNOA | 1BOEA | 1BORA | 1BOUA | 1BOUB |
| 1BPBA | 1BQBA | 1BR0A | 1BRTA | 1BRZA |
| 1BSHA | 1BT3A | 1BUYA | 1BW0A | 1BX7A |
| 1BXEA | 1BY1A | 1BYIA | 1BYYA | 1BZKA |
| 1C01A | 1C17M | 1C1DA | 1C3CA | 1C3YA |
| 1C4QA | 1C5EA | 1C7KA | 1C8PA | 1C9KA |
| 1CB8A | 1CC8A | 1CCWA | 1CCWB | 1CEJA |
| 1CEWI | 1CF4B | 1CFEA | 1CFRA | 1CHDA |
| 1CHMA | 1CIXA | 1CIYA | 1CJWA | 1CL4A |
| 1CMCA | 1CNOA | 1CNT1 | 1CO4A | 1COJA |
| 1COLA | 1COUA | 1CQXA | 1CREA | 1CS1A |
| 1CT9A | 1CTFA | 1CTOA | 1CV8A | 1CVRA |
| 1CWXA | 1CX1A | 1CX4A | 1CXPA | 1CXQA |
| 1CY5A | 1CYWA | 1D0DA | 1D1DA | 1D2PA |
| 1D3YA | 1D4BA | 1D4OA | 1D4TA | 1D5TA |
| 1D6BA | 1D6GA | 1D8BA | 1D8JA | 1D8WA |
| 1DBWA | 1DCSA | 1DDFA | 1DE3A | 1DG6A |
| 1DGWA | 1DGWX | 1DGWY | 1DHNA | 1DHRA |
| 1DHSA | 1DIPA | 1DJ0A | 1DK8A | 1DLFL |
| 1DLYA | 1DMTA | 1DNYA | 1DOWA | 1DP4A |
| 1DP5B | 1DQ3A | 1DQCA | 1DQGA | 1DQSA |
| 1DQZA | 1DROA | 1DRWA | 1DS1A | 1DTDB |
| 1DUNA | 1DUVG | 1DV0A | 1DV5A | 1DVOA |
| 1DWKA | 1DX0A | 1DX5I | 1DY5A | 1DYLA |
| 1DYNA | 1DYOA | 1DZ7A | 1E0EA | 1E17A |
| 1E19A | 1E1HB | 1E29A | 1E2WA | 1E42A |
| 1E44A | 1E44B | 1E52A | 1E58A | 1E5KA |
| 1E5UI | 1E6UA | 1E7LA | 1E8RA | 1EAIC |
| 1EAQA | 1EB6A | 1ECAA | 1ECFA | 1ED7A |
| 1EDXA | 1EEXA | 1EEXB | 1EEXG | 1EF8A |
| 1EFUB | 1EHSA | 1EHXA | 1EI1A | 1EI5A |
| 1EI7A | 1EIWA | 1EJ5A | 1EJ8A | 1EJDA |
| 1ELWA | 1EMWA | 1EN2A | 1ENWA | 1EOKA |
| 1EP3B | 1EQ1A | 1ERDA | 1ES9A | 1ESCA |
| 1ESIA | 1ESOA | 1ESXA | 1EU1A | 1EU8A |
| 1EUDA | 1EUDB | 1EUVA | 1EUWA | 1EVFA |
| 1EVXA | 1EW4A | 1EWFA | 1EWKA | 1EYBA |
| 1EYRA | 1EYVA | 1EZGA | 1EZTA | 1EZWA |
| 1F0LA | 1F1EA | 1F1JA | 1F20A | 1F2HA |
| 1F2TA | 1F2TB | 1F46A | 1F4PA | 1F53A |
| 1F5VA | 1F5XA | 1F60B | 1F61A | 1F6VA |
| 1F76A | 1F86A | 1F8EA | 1F94A | 1F9MA |
| 1FADA | 1FAFA | 1FC3A | 1FC6A | 1FCQA |
| 1FCYA | 1FD3A | 1FE6A | 1FEXA | 1FFGB |
| 1FHOA | 1FIPA | 1FJ2A | 1FJHA | 1FK5A |
| 1FKMA | 1FLEI | 1FM0D | 1FM0E | 1FN9A |
| 1FO8A | 1FSBA | 1FSGA | 1FSHA | 1FT5A |
| 1FTRA | 1FVIA | 1FX2A | 1FXOA | 1FYEA |
| 1G03A | 1G0SA | 1G25A | 1G2HA | 1G2IA |
| 1G2RA | 1G3PA | 1G5AA | 1G5HA | 1G60A |
| 1G61A | 1G66A | 1G6EA | 1G6XA | 1G6ZA |
| 1G7DA | 1G8KA | 1G8MA | 1G8QA | 1G9PA |
| 1GA6A | 1GA8A | 1GCIA | 1GE7A | 1GE9A |
| 1GH9A | 1GHHA | 1GJJA | 1GK9A | 1GK9B |
| 1GKGA | 1GKMA | 1GKPA | 1GL2A | 1GL2B |
| 1GL2C | 1GL2D | 1GMXA | 1GNLA | 1GNYA |
| 1GO9A | 1GP6A | 1GP8A | 1GPLA | 1GPPA |
| 1GPQA | 1GPRA | 1GQ8A | 1GQIA | 1GSAA |
| 1GSJA | 1GU2A | 1GU7A | 1GUIA | 1GUQA |
| 1GUTA | 1GV9A | 1GVCA | 1GVDA | 1GVFA |
| 1GVNA | 1GVNB | 1GWEA | 1GWMA | 1GWUA |
| 1GXMA | 1GXUA | 1GXYA | 1GYFA | 1GYXA |
| 1GYZA | 1H0AA | 1H0ZA | 1H16A | 1H1NA |
| 1H21A | 1H2KA | 1H2KS | 1H32A | 1H3ZA |
| 1H4RA | 1H4XA | 1H54A | 1H5PA | 1H6DA |
| 1H6GA | 1H72C | 1H7DA | 1H8BA | 1H8BB |
| 1H8MA | 1H97A | 1H9AA | 1HA8A | 1HANA |
| 1HBNA | 1HBNB | 1HBNC | 1HBWA | 1HCDA |
| 1HDHA | 1HDOA | 1HE1A | 1HF9A | 1HFES |
| 1HGVA | 1HH8A | 1HHNA | 1HJSA | 1HK6A |
| 1HLBA | 1HM9A | 1HN0A | 1HN3A | 1HN6A |
| 1HNFA | 1HP1A | 1HP8A | 1HPIA | 1HQ0A |
| 1HS7A | 1HTRP | 1HUUA | 1HUWA | 1HW1A |
| 1HX2A | 1HX6A | 1HXIA | 1HXNA | 1HY9A |
| 1HYOA | 1HYPA | 1HYWA | 1HZ4A | 1HZ6A |
| 1HZEA | 1HZTA | 1I0SA | 1I17A | 1I1SA |
| 1I1WA | 1I24A | 1I27A | 1I2TA | 1I2UA |
| 1I35A | 1I4UA | 1I4VA | 1I5JA | 1I5PA |
| 1I6ZA | 1I71A | 1I7QA | 1I7QB | 1I88A |
| 1I8OA | 1I9SA | 1I9ZA | 1IB8A | 1ICFI |
| 1ID0A | 1IDPA | 1IFPA | 1IG6A | 1IGQA |
| 1IHGA | 1IHVA | 1IIBA | 1IIEA | 1IIOA |
| 1IJBA | 1IM8A | 1IN0A | 1INLA | 1IOJA |
| 1IOMA | 1IP9A | 1IPCA | 1IQOA | 1IQVA |
| 1IQZA | 1IRZA | 1ISPA | 1ISUA | 1IT3A |
| 1ITHA | 1ITXA | 1IUFA | 1IUHA | 1IV8A |
| 1IW4A | 1IX9A | 1IXHA | 1IYRA | 1IZ6A |
| 1IZMA | 1J0PA | 1J0TA | 1J1HA | 1J1TA |
| 1J27A | 1J2JB | 1J2MA | 1J30A | 1J34C |
| 1J57A | 1J58A | 1J5WA | 1J5YA | 1J6PA |
| 1J6QA | 1J7GA | 1J7MA | 1J83A | 1J8BA |
| 1J98A | 1J9IA | 1JB0A | 1JB0D | 1JB0E |
| 1JB0F | 1JB0J | 1JB0K | 1JB0L | 1JB3A |
| 1JBEA | 1JBIA | 1JBMA | 1JCDA | 1JDQA |
| 1JDWA | 1JEIA | 1JF3A | 1JFBA | 1JG1A |
| 1JH3A | 1JH6A | 1JHGA | 1JI1A | 1JI7A |
| 1JIGA | 1JIWI | 1JIXA | 1JJDA | 1JJGA |
| 1JLYA | 1JM0A | 1JM1A | 1JNDA | 1JNIA |
| 1JO0A | 1JO6A | 1JP4A | 1JQ5A | 1JR5A |
| 1JR6A | 1JR7A | 1JRMA | 1JSGA | 1JSUC |
| 1JT8A | 1JUHA | 1JUNA | 1JVAA | 1JW3A |
| 1JWEA | 1JXCA | 1JY1A | 1JY3N | 1JY3O |
| 1JY3P | 1JYHA | 1JYTA | 1JZ8A | 1JZTA |
| 1JZUA | 1K07A | 1K0HA | 1K0MA | 1K0ZA |
| 1K1VA | 1K20A | 1K2XA | 1K2XB | 1K30A |
| 1K36A | 1K3IA | 1K3YA | 1K4IA | 1K5CA |
| 1K5KA | 1K5NA | 1K5NB | 1K6ZA | 1K75A |
| 1K77A | 1K7CA | 1K7JA | 1K8VA | 1K92A |
| 1KA1A | 1KBEA | 1KBHB | 1KCQA | 1KDKA |
| 1KG1A | 1KGDA | 1KGSA | 1KI0A | 1KIDA |
| 1KJ6A | 1KJKA | 1KJQA | 1KJSA | 1KKOA |
| 1KL9A | 1KLOA | 1KMTA | 1KMVA | 1KMXA |
| 1KN6A | 1KN7A | 1KNGA | 1KNYA | 1KOEA |
| 1KOLA | 1KP6A | 1KPTA | 1KQ6A | 1KQFA |
| 1KQFB | 1KQFC | 1KQPA | 1KQRA | 1KS8A |
| 1KSIA | 1KSQA | 1KT6A | 1KTGA | 1KV4A |
| 1KVEA | 1KVEB | 1KVNA | 1KWFA | 1KX2A |
| 1KXLA | 1KYFA | 1KZLA | 1L2MA | 1L3KA |
| 1L3YA | 1L5CA | 1L5JA | 1L6PA | 1L6RA |
| 1L7AA | 1L7YA | 1L8YA | 1L9LA | 1L9XA |
| 1LB3A | 1LBAA | 1LBWA | 1LC5A | 1LEAA |
| 1LENA | 1LF6A | 1LFWA | 1LHPA | 1LKEA |
| 1LKIA | 1LKKA | 1LL2A | 1LLFA | 1LMIA |
| 1LMLA | 1LNIA | 1LNSA | 1LO7A | 1LPBA |
| 1LPEA | 1LPVA | 1LQ7A | 1LQTA | 1LQVA |
| 1LR0A | 1LR1A | 1LR5A | 1LRZA | 1LS1A |
| 1LS4A | 1LSHA | 1LSHB | 1LTSC | 1LTZA |
| 1LU4A | 1LUCA | 1LV3A | 1LW1A | 1LWBA |
| 1LYBA | 1LYBB | 1LYVA | 1LZLA | 1M0KA |
| 1M0WA | 1M15A | 1M1FA | 1M1QA | 1M1ZA |
| 1M22A | 1M2DA | 1M2EA | 1M40A | 1M48A |
| 1M4IA | 1M4LA | 1M4YA | 1M55A | 1M5TA |
| 1M65A | 1M6SA | 1M70A | 1M7KA | 1M93A |
| 1M93B | 1M93C | 1M9LA | 1M9OA | 1M9ZA |
| 1MBAA | 1MC2A | 1MFNA | 1MG4A | 1MIXA |
| 1MJ4A | 1MJ5A | 1MJNA | 1MJSA | 1MJUL |
| 1MK0A | 1MKKA | 1MMLA | 1MN8A | 1MNTA |
| 1MO0A | 1MOLA | 1MP1A | 1MRAA | 1MSCA |
| 1MSKA | 1MSPA | 1MTPA | 1MTPB | 1MTYB |
| 1MTYD | 1MTYG | 1MUNA | 1MUWA | 1MV3A |
| 1MV8A | 1MVLA | 1MW4A | 1MWQA | 1MWWA |
| 1MXRA | 1MYNA | 1N0QA | 1N0ZA | 1N13A |
| 1N13B | 1N1FA | 1N2ZA | 1N31A | 1N3GA |
| 1N3JA | 1N3LA | 1N40A | 1N4CA | 1N4WA |
| 1N62A | 1N62B | 1N62C | 1N6ZA | 1N7HA |
| 1N7LA | 1N8VA | 1N91A | 1N93X | 1NARA |
| 1NB9A | 1NC5A | 1NCSA | 1ND9A | 1NE5A |
| 1NEIA | 1NEPA | 1NEQA | 1NFAA | 1NFPA |
| 1NG7A | 1NH8A | 1NI7A | 1NKDA | 1NKGA |
| 1NKIA | 1NKZA | 1NKZB | 1NNFA | 1NNHA |
| 1NNVA | 1NNXA | 1NPSA | 1NQ4A | 1NQJA |
| 1NRJA | 1NRJB | 1NS5A | 1NSJA | 1NSZA |
| 1NTHA | 1NTYA | 1NU0A | 1NULA | 1NUYA |
| 1NVUS | 1NWWA | 1NWZA | 1NXIA | 1NXMA |
| 1NXUA | 1NY4A | 1NY9A | 1NYNA | 1NYOA |
| 1NZ0A | 1NZ8A | 1NZ9A | 1NZJA | 1O1MA |
| 1O20A | 1O22A | 1O2DA | 1O4WA | 1O51A |
| 1O54A | 1O57A | 1O59A | 1O69A | 1O6AA |
| 1O6VA | 1O7IA | 1O7JA | 1O83A | 1O8RA |
| 1O91A | 1O9IA | 1O9YA | 1OAIA | 1OAOC |
| 1OBOA | 1OBPA | 1OC0B | 1OCYA | 1OD3A |
| 1ODMA | 1OEDC | 1OEJA | 1OEWA | 1OF1A |
| 1OF8A | 1OF9A | 1OFDA | 1OFLA | 1OFWA |
| 1OFZA | 1OGOX | 1OGQA | 1OH1A | 1OH4A |
| 1OHVA | 1OI7A | 1OIHA | 1OJ5A | 1OJ7A |
| 1OJHA | 1OK0A | 1OKSA | 1OL5B | 1OLTA |
| 1ON4A | 1ON6A | 1ONVB | 1ONWA | 1OQ1A |
| 1OR4A | 1OR7C | 1ORJA | 1ORMA | 1ORUA |
| 1OSXA | 1OSYA | 1OT1A | 1OTGA | 1OTKA |
| 1OTRA | 1OUNA | 1OUWA | 1OV2A | 1OVYA |
| 1OWLA | 1OWXA | 1OXXK | 1OZ2A | 1P0HA |
| 1P0ZA | 1P1DA | 1P1JA | 1P1XA | 1P32A |
| 1P35A | 1P3CA | 1P3DA | 1P4CA | 1P4QA |
| 1P4WA | 1P5DX | 1P5SA | 1P5XA | 1P5ZB |
| 1P6OA | 1P6RA | 1P8AA | 1P90A | 1P94A |
| 1P99A | 1P9GA | 1P9KA | 1P9YA | 1PA4A |
| 1PBEA | 1PBJA | 1PBUA | 1PBWA | 1PBYA |
| 1PBYB | 1PBYC | 1PC2A | 1PCFA | 1PDOA |
| 1PF5A | 1PFBA | 1PFSA | 1PG4A | 1PGYA |
| 1PIIA | 1PKHA | 1PL3A | 1PL8A | 1PLQA |
| 1PM4A | 1PM6A | 1PMMA | 1PMSA | 1PN4A |
| 1POCA | 1POIA | 1POIB | 1POUA | 1PPRM |
| 1PQ7A | 1PQHA | 1PQXA | 1PRQA | 1PSRA |
| 1PSYA | 1PT6A | 1PU1A | 1PU6A | 1PUCA |
| 1PULA | 1PV0A | 1PVMA | 1PWAA | 1PX0A |
| 1PX5A | 1PXVC | 1PXZA | 1PYAA | 1PYAB |
| 1PYTA | 1PYVA | 1PZ4A | 1PZQA | 1Q08A |
| 1Q1OA | 1Q1VA | 1Q2ZA | 1Q35A | 1Q4UA |
| 1Q55A | 1Q5FA | 1Q5LA | 1Q5YA | 1Q5ZA |
| 1Q6AA | 1Q6OA | 1Q6ZA | 1Q74A | 1Q7EA |
| 1Q7FA | 1Q7LA | 1Q7LB | 1Q8DA | 1Q8IA |
| 1Q8KA | 1Q9UA | 1QA5A | 1QAZA | 1QCKA |
| 1QD1A | 1QDDA | 1QF8A | 1QFTA | 1QG8A |
| 1QGIA | 1QHDA | 1QHKA | 1QI9A | 1QJPA |
| 1QKLA | 1QKRA | 1QKSA | 1QL1A | 1QLWA |
| 1QMJA | 1QMYA | 1QNRA | 1QO0A | 1QO0D |
| 1QOPA | 1QOPB | 1QOYA | 1QPOA | 1QQFA |
| 1QQGA | 1QQP1 | 1QQP2 | 1QQP3 | 1QQP4 |
| 1QQRA | 1QSAA | 1QTOA | 1QU5A | 1QV1A |
| 1QW9A | 1QWGA | 1QWOA | 1QWRA | 1QWYA |
| 1QXFA | 1QYAA | 1QZMA | 1QZRA | 1R05A |
| 1R0MA | 1R0UA | 1R0VA | 1R29A | 1R3BA |
| 1R3SA | 1R45A | 1R4GA | 1R4KA | 1R4XA |
| 1R5EA | 1R5LA | 1R5MA | 1R5SA | 1R5ZA |
| 1R6CX | 1R6DA | 1R6JA | 1R6RA | 1R6WA |
| 1R6XA | 1R79A | 1R7AA | 1R89A | 1R8GA |
| 1R8HA | 1R8OA | 1R8OB | 1R8SE | 1R9LA |
| 1RA0A | 1RAXA | 1RCQA | 1RCWA | 1RDQE |
| 1REGX | 1RESA | 1RF8B | 1RFXA | 1RG8A |
| 1RHCA | 1RHSA | 1RI9A | 1RIPA | 1RKIA |
| 1RL0A | 1RLIA | 1RLYA | 1RM6B | 1RMGA |
| 1RO5A | 1ROCA | 1RP0A | 1RQ6A | 1RQBA |
| 1RRZA | 1RSOA | 1RSOB | 1RTQA | 1RU4A |
| 1RUTX | 1RV9A | 1RVEA | 1RW1A | 1RW2A |
| 1RWHA | 1RWIA | 1RWJA | 1RWRA | 1RX0A |
| 1RXQA | 1RXZA | 1RY3A | 1RY9A | 1RYJA |
| 1RYKA | 1RYQA | 1S05A | 1S0IA | 1S1DA |
| 1S1QA | 1S3AA | 1S3CA | 1S4CA | 1S4KA |
| 1S4NA | 1S5DA | 1S6YA | 1S7AA | 1S7EA |
| 1S7ZA | 1S96A | 1S99A | 1S9RA | 1S9UA |
| 1SAUA | 1SB0A | 1SBPA | 1SBXA | 1SBYA |
| 1SCFA | 1SDIA | 1SDWA | 1SE7A | 1SEDA |
| 1SENA | 1SFPA | 1SFXA | 1SG4A | 1SG5A |
| 1SG7A | 1SGOA | 1SH8A | 1SHUX | 1SJWA |
| 1SLUA | 1SMXA | 1SO9A | 1SQ9A | 1SQRA |
| 1SQSA | 1SR4A | 1SRAA | 1SRVA | 1SS3A |
| 1SS6A | 1SSEB | 1SSKA | 1SSLA | 1STAA |
| 1SU8A | 1SVBA | 1SVFA | 1SVSA | 1SVVA |
| 1SX7A | 1SXMA | 1SZ2A | 1SZLA | 1SZVA |
| 1SZWA | 1T07A | 1T0BA | 1T0GA | 1T0HB |
| 1T0TV | 1T0YA | 1T17A | 1T1UA | 1T23A |
| 1T2DA | 1T2WA | 1T3KA | 1T3OA | 1T3YA |
| 1T4NA | 1T4YA | 1T50A | 1T5OA | 1T61A |
| 1T6AA | 1T6CA | 1T6UA | 1T6WA | 1T82A |
| 1T8KA | 1T92A | 1T9HA | 1TAFA | 1TBAA |
| 1TBFA | 1TDHA | 1TDPA | 1TE2A | 1TFBA |
| 1TH5A | 1TI5A | 1TIFA | 1TIIC | 1TITA |
| 1TJFA | 1TJVA | 1TJXA | 1TJYA | 1TK9A |
| 1TKEA | 1TKNA | 1TL2A | 1TM9A | 1TNSA |
| 1TOAA | 1TP6A | 1TPGA | 1TQGA | 1TQJA |
| 1TQZA | 1TR0A | 1TT8A | 1TTYA | 1TTZA |
| 1TU9A | 1TUHA | 1TUJA | 1TUKA | 1TULA |
| 1TUWA | 1TUZA | 1TVCA | 1TVIA | 1TVMA |
| 1TVNA | 1TVXA | 1TWDA | 1TX2A | 1TXGA |
| 1TXKA | 1TZPA | 1TZYB | 1TZYC | 1U02A |
| 1U07A | 1U14A | 1U19A | 1U2HA | 1U36A |
| 1U5DA | 1U5KA | 1U5MA | 1U5PA | 1U60A |
| 1U69A | 1U6EA | 1U6KA | 1U6TA | 1U7GA |
| 1U7IA | 1U89A | 1U8VA | 1U94A | 1U96A |
| 1U9DA | 1UA4A | 1UB1A | 1UCDA | 1UCRA |
| 1UCSA | 1UDKA | 1UEKA | 1UEOA | 1UF5A |
| 1UFGA | 1UFMA | 1UFOA | 1UFWA | 1UFYA |
| 1UFZA | 1UG1A | 1UG2A | 1UG7A | 1UG8A |
| 1UGHI | 1UGJA | 1UGLA | 1UGXA | 1UI0A |
| 1UILA | 1UJ2A | 1UJ8A | 1UJLA | 1UJOA |
| 1UK8A | 1UKKA | 1UL4A | 1UMQA | 1UNNC |
| 1UNQA | 1UOYA | 1UOZA | 1UPGA | 1UPSA |
| 1UQVA | 1URFA | 1URQA | 1URQC | 1US0A |
| 1US5A | 1US7B | 1USEA | 1USGA | 1USTA |
| 1UT1A | 1UTAA | 1UUFA | 1UUJA | 1UUQA |
| 1UUYA | 1UV4A | 1UV7A | 1UWCA | 1UWKA |
| 1UX6A | 1UXOA | 1UZ3A | 1V05A | 1V0AA |
| 1V0WA | 1V1CA | 1V2BA | 1V2YA | 1V32A |
| 1V4RA | 1V5IB | 1V5MA | 1V5NA | 1V5RA |
| 1V5VA | 1V65A | 1V66A | 1V6PA | 1V6ZA |
| 1V74A | 1V74B | 1V7WA | 1V7ZA | 1V82A |
| 1V8DA | 1V93A | 1V95A | 1V9VA | 1V9WA |
| 1V9YA | 1VA0A | 1VA6A | 1VBWA | 1VCLA |
| 1VD4A | 1VDFA | 1VDIA | 1VDKA | 1VDLA |
| 1VDYA | 1VE9A | 1VEEA | 1VEJA | 1VFIA |
| 1VG0A | 1VH5A | 1VHIA | 1VHNA | 1VIBA |
| 1VIMA | 1VJGA | 1VJNA | 1VJUA | 1VJVA |
| 1VK1A | 1VK3A | 1VK4A | 1VKEA | 1VKFA |
| 1VKHA | 1VKIA | 1VKKA | 1VKMA | 1VKRA |
| 1VKYA | 1VL1A | 1VL7A | 1VLAA | 1VLBA |
| 1VLKA | 1VLSA | 1VLYA | 1VMBA | 1VMGA |
| 1VMHA | 1VMJA | 1VMOA | 1VP8A | 1VPBA |
| 1VPDA | 1VPKA | 1VPMA | 1VPRA | 1VPUA |
| 1VQ0A | 1VQ3A | 1VQBA | 1VQSA | 1VR7A |
| 1VR8A | 1VRAA | 1VRAB | 1VS0A | 1VSRA |
| 1VYBA | 1VYIA | 1VYKA | 1VYNA | 1VYRA |
| 1VZMA | 1VZYA | 1W0BA | 1W0HA | 1W0MA |
| 1W0NA | 1W1HA | 1W1OA | 1W23A | 1W2QA |
| 1W2WA | 1W2WB | 1W3EX | 1W53A | 1W55A |
| 1W5QA | 1W5RA | 1W66A | 1W6KA | 1W6SA |
| 1W6SB | 1W73A | 1W7CA | 1W85I | 1W8SA |
| 1W8UA | 1W91A | 1W94A | 1W96A | 1W9HA |
| 1W9IA | 1W9RA | 1WB4A | 1WBAA | 1WBHA |
| 1WC1A | 1WC2A | 1WCKA | 1WCLA | 1WCWA |
| 1WD3A | 1WDCA | 1WDCB | 1WDCC | 1WDDA |
| 1WDDS | 1WDJA | 1WDVA | 1WE6A | 1WEHA |
| 1WELA | 1WEOA | 1WEQA | 1WEYA | 1WFEA |
| 1WFLA | 1WFMA | 1WFRA | 1WFYA | 1WGRA |
| 1WGSA | 1WGWA | 1WH2A | 1WH5A | 1WH9A |
| 1WHBA | 1WHIA | 1WHRA | 1WHSA | 1WHSB |
| 1WHUA | 1WHVA | 1WHXA | 1WHZA | 1WI1A |
| 1WI5A | 1WI9A | 1WICA | 1WIDA | 1WIGA |
| 1WIHA | 1WIJA | 1WILA | 1WIMA | 1WINA |
| 1WIRA | 1WIXA | 1WJ5A | 1WJPA | 1WJRA |
| 1WJTA | 1WJVA | 1WJWA | 1WK0A | 1WK1A |
| 1WKAA | 1WKIA | 1WKQA | 1WKTA | 1WKUA |
| 1WLGA | 1WLOA | 1WLUA | 1WLXA | 1WLZA |
| 1WMTA | 1WN2A | 1WNYA | 1WOLA | 1WPIA |
| 1WPNA | 1WQJI | 1WQKA | 1WQLB | 1WS8A |
| 1WT7A | 1WTJA | 1WU3I | 1WU9A | 1WUIL |
| 1WUIS | 1WUWA | 1WV3A | 1WVFA | 1WVKA |
| 1WWIA | 1WWTA | 1WWUA | 1WWWV | 1WWWX |
| 1WWYA | 1WXOA | 1WYJA | 1WYMA | 1WYOA |
| 1WZDA | 1X0HA | 1X1NA | 1X3AA | 1X3BA |
| 1X3CA | 1X3QA | 1X4PA | 1X4RA | 1X4SA |
| 1X4TA | 1X51A | 1X52A | 1X53A | 1X54A |
| 1X5MA | 1X65A | 1X67A | 1X6FA | 1X6GA |
| 1X6IA | 1X6MA | 1X6OA | 1X6ZA | 1X7DA |
| 1X82A | 1X8LA | 1X8QA | 1X91A | 1X9BA |
| 1X9IA | 1X9LA | 1XBIA | 1XCRA | 1XD3A |
| 1XDNA | 1XEEA | 1XFFA | 1XG0A | 1XG0C |
| 1XG4A | 1XGKA | 1XHNA | 1XHSA | 1XI7A |
| 1XJCA | 1XJHA | 1XJSA | 1XJUA | 1XKIA |
| 1XKSA | 1XL3A | 1XL3C | 1XLQA | 1XMKA |
| 1XMTA | 1XMXA | 1XN5A | 1XOCA | 1XODA |
| 1XOVA | 1XPAA | 1XPIA | 1XPWA | 1XQOA |
| 1XRDA | 1XSOA | 1XSVA | 1XSXA | 1XT5A |
| 1XTEA | 1XTTA | 1XU1R | 1XU2R | 1XU6A |
| 1XUBA | 1XV5A | 1XVSA | 1XWEA | 1XWYA |
| 1Y0HA | 1Y0KA | 1Y0PA | 1Y0UA | 1Y12A |
| 1Y1OA | 1Y43B | 1Y63A | 1Y66A | 1Y6DA |
| 1Y6UA | 1Y6XA | 1Y7JA | 1Y7RA | 1Y7TA |
| 1Y7XA | 1Y93A | 1Y9JA | 1YACA | 1YARA |
| 1YARH | 1YARO | 1YC9A | 1YD0A | 1YDGA |
| 1YDUA | 1YDXA | 1YDYA | 1YE8A | 1YFBA |
| 1YFQA | 1YG9A | 1YGMA | 1YGTA | 1YHPA |
| 1YHTA | 1YISA | 1YJ7A | 1YKDA | 1YKIA |
| 1YKSA | 1YLEA | 1YLLA | 1YLOA | 1YM3A |
| 1YNPA | 1YO4A | 1YOCA | 1YOYA | 1YPHC |
| 1YPHE | 1YQGA | 1YQHA | 1YQSA | 1YR1A |
| 1YRBA | 1YRKA | 1YRRA | 1YS3A | 1YSQA |
| 1YSYA | 1YT3A | 1YT8A | 1YTLA | 1YTVM |
| 1YU0A | 1YU5X | 1YUAA | 1YUMA | 1YVCA |
| 1YWFA | 1YWMA | 1YWXA | 1YWYA | 1YX1A |
| 1YX4A | 1YYCA | 1YYPA | 1YZ1A | 1YZFA |
| 1YZHA | 1YZSA | 1YZVA | 1Z0NA | 1Z0WA |
| 1Z1DA | 1Z1ZA | 1Z2FA | 1Z2NX | 1Z2UA |
| 1Z2WA | 1Z3EB | 1Z3XA | 1Z40A | 1Z4HA |
| 1Z4RA | 1Z5FA | 1Z60A | 1Z67A | 1Z6BA |
| 1Z6MA | 1Z6NA | 1Z6OA | 1Z70X | 1Z72A |
| 1Z7AA | 1Z7Z2 | 1Z7ZI | 1Z8GA | 1Z8SA |
| 1Z96A | 1Z9BA | 1ZAEA | 1ZARA | 1ZBFA |
| 1ZC1A | 1ZCEA | 1ZD7A | 1ZE3H | 1ZEJA |
| 1ZELA | 1ZG2A | 1ZGKA | 1ZHCA | 1ZHSA |
| 1ZI8A | 1ZJCA | 1ZJJA | 1ZK4A | 1ZK5A |
| 1ZK8A | 1ZKEA | 1ZKUA | 1ZL0A | 1ZL8A |
| 1ZL8B | 1ZLDA | 1ZMAA | 1ZNNA | 1ZO0A |
| 1ZOVA | 1ZPVA | 1ZR6A | 1ZR9A | 1ZRNA |
| 1ZRRA | 1ZRUA | 1ZRXA | 1ZSOA | 1ZTSA |
| 1ZU1A | 1ZU2A | 1ZUD1 | 1ZUUA | 1ZVTA |
| 1ZW8A | 1ZWTA | 1ZX2A | 1ZX8A | 1ZXAA |
| 1ZXFA | 1ZY7A | 1ZY9A | 1ZYIA | 1ZZ1A |
| 1ZZAA | 1ZZKA | 1ZZMA | 2A05A | 2A14A |
| 2A15A | 2A1VA | 2A26A | 2A2MA | 2A2PA |
| 2A35A | 2A3DA | 2A3NA | 2A4DA | 2A4HA |
| 2A50A | 2A5HA | 2A5ZA | 2A65A | 2A67A |
| 2A6AA | 2A6CA | 2A6SA | 2A6ZA | 2A72A |
| 2A7OA | 2A7YA | 2A9IA | 2A9SA | 2AALA |
| 2ABKA | 2ABOA | 2ABSA | 2ABWA | 2ACFA |
| 2ACMB | 2ADLA | 2ADVC | 2ADZA | 2AEBA |
| 2AFBA | 2AFFB | 2AFHE | 2AG4A | 2AGAA |
| 2AGKA | 2AGMA | 2AH5A | 2AHFA | 2AHQA |
| 2AHRA | 2AHUA | 2AI6A | 2AIBA | 2AIVA |
| 2AJ6A | 2AJ7A | 2AKAB | 2AKKA | 2AKOA |
| 2AKZA | 2AL3A | 2ALCA | 2AMHA | 2AMLA |
| 2AN7A | 2ANUA | 2ANXA | 2APJA | 2APLA |
| 2AQ6A | 2AQAA | 2AQXA | 2ARCA | 2ARTA |
| 2ARWA | 2AVXA | 2AXCA | 2AXLA | 2AXWA |
| 2AYAA | 2AYDA | 2AYJA | 2AZ4A | 2AZWA |
| 2B06A | 2B0AA | 2B0HA | 2B0TA | 2B0VA |
| 2B1WA | 2B2NA | 2B3FA | 2B3HA | 2B3WA |
| 2B4AA | 2B4JC | 2B4VA | 2B4WA | 2B5WA |
| 2B61A | 2B68A | 2B6CA | 2B81A | 2B82A |
| 2B8MA | 2B8WA | 2B97A | 2B9KA | 2B9ZA |
| 2BA3A | 2BAFA | 2BAYA | 2BBAA | 2BBHA |
| 2BCMA | 2BD0A | 2BDRA | 2BE3A | 2BEKA |
| 2BF6A | 2BFDA | 2BFDB | 2BFWA | 2BG9A |
| 2BGOA | 2BH1A | 2BH1X | 2BHUA | 2BIBA |
| 2BICA | 2BIDA | 2BIVA | 2BIWA | 2BJFA |
| 2BJIA | 2BJNA | 2BJQA | 2BJVA | 2BK8A |
| 2BK9A | 2BKDN | 2BKFA | 2BKRA | 2BKXA |
| 2BKYA | 2BL2A | 2BL5A | 2BLFB | 2BLNA |
| 2BM5A | 2BM8A | 2BMOA | 2BMOB | 2BN8A |
| 2BNLA | 2BNMA | 2BNXA | 2BO9B | 2BOGX |
| 2BPTA | 2BRFA | 2BRYA | 2BS3C | 2BSJA |
| 2BT9A | 2BU3A | 2BUEA | 2BV2A | 2BVBA |
| 2BVFA | 2BW2A | 2BW3A | 2BW3B | 2BW4A |
| 2BWFA | 2BWRA | 2BX6A | 2BYOA | 2BYUA |
| 2BYZA | 2BZ1A | 2BZVA | 2C0DA | 2C0NA |
| 2C0SA | 2C0WA | 2C1IA | 2C1LA | 2C1VA |
| 2C2PA | 2C2UA | 2C36A | 2C3WA | 2C42A |
| 2C43A | 2C4WA | 2C4XA | 2C52B | 2C55A |
| 2C5AA | 2C5KT | 2C5LC | 2C5UA | 2C60A |
| 2C61A | 2C6QA | 2C71A | 2C78A | 2C7CO |
| 2C7HA | 2C7NA | 2C92A | 2C9GA | 2C9QA |
| 2C9WA | 2CA5A | 2CA6A | 2CALA | 2CARA |
| 2CAYA | 2CB8A | 2CBZA | 2CCMA | 2CCQA |
| 2CCVA | 2CD7A | 2CDUA | 2CE2X | 2CE8A |
| 2CEVA | 2CFUA | 2CG7A | 2CGTO | 2CH0A |
| 2CH5A | 2CH8A | 2CHCA | 2CHHA | 2CHOA |
| 2CI1A | 2CISA | 2CIUA | 2CIWA | 2CJ4A |
| 2CJSA | 2CJSC | 2CJTA | 2CKAA | 2CKKA |
| 2CKLB | 2CKNA | 2CMZA | 2CN3A | 2CNQA |
| 2CO3A | 2CO5A | 2CO8A | 2CO9A | 2COBA |
| 2COCA | 2COKA | 2CONA | 2COSA | 2COVD |
| 2CP9A | 2CPIA | 2CPRA | 2CQ2A | 2CQ8A |
| 2CQAA | 2CQJA | 2CQKA | 2CQLA | 2CQMA |
| 2CQOA | 2CQYA | 2CR2A | 2CR4A | 2CR8A |
| 2CR9A | 2CRBA | 2CRIA | 2CRQA | 2CRUA |
| 2CRVA | 2CS2A | 2CS7A | 2CS8A | 2CSNA |
| 2CSOA | 2CSZA | 2CTQA | 2CTUA | 2CUDA |
| 2CUJA | 2CULA | 2CVDA | 2CW1A | 2CWRA |
| 2CWSA | 2CWYA | 2CX1A | 2CXAA | 2CXCA |
| 2CXHA | 2CXIA | 2CXNA | 2CXYA | 2CYJA |
| 2CZ4A | 2CZLA | 2CZOA | 2CZRA | 2D0OA |
| 2D0OB | 2D0WA | 2D1FA | 2D1LA | 2D1SA |
| 2D1UA | 2D28C | 2D46A | 2D4PA | 2D56A |
| 2D58A | 2D5BA | 2D5MA | 2D5WA | 2D81A |
| 2D85A | 2D8BA | 2D8QA | 2D8RA | 2D8SA |
| 2D8VA | 2D96A | 2D9DA | 2D9KA | 2D9RA |
| 2D9ZA | 2DA4A | 2DAEA | 2DALA | 2DAQA |
| 2DASA | 2DAVA | 2DAWA | 2DB2A | 2DB7A |
| 2DBBA | 2DBGA | 2DBHA | 2DBJA | 2DBYA |
| 2DCEA | 2DCKA | 2DCVA | 2DDHA | 2DDXA |
| 2DE3A | 2DE6A | 2DEFA | 2DEJA | 2DG1A |
| 2DGYA | 2DGZA | 2DH2A | 2DHXA | 2DHYA |
| 2DHZA | 2DI0A | 2DI7A | 2DIPA | 2DIRA |
| 2DIWA | 2DJ0A | 2DJKA | 2DJPA | 2DJRA |
| 2DJVA | 2DK4A | 2DKAA | 2DKJA | 2DKLA |
| 2DKOA | 2DKOB | 2DKTA | 2DKVA | 2DKYA |
| 2DLBA | 2DLEA | 2DLQA | 2DMFA | 2DMHA |
| 2DMWA | 2DNEA | 2DNFA | 2DNXA | 2DO3A |
| 2DO9A | 2DOFA | 2DQAA | 2DS5A | 2DSKA |
| 2DSMA | 2DSXA | 2DSYA | 2DT4A | 2DT8A |
| 2DTJA | 2DTRA | 2DULA | 2DUNA | 2DUYA |
| 2DVHA | 2DVMA | 2DW0A | 2DW5A | 2DWUA |
| 2DXAA | 2DY0A | 2DY1A | 2DY8A | 2DYIA |
| 2DYJA | 2DZLA | 2DZMA | 2E01A | 2E0AA |
| 2E0GA | 2E11A | 2E2AA | 2E2FA | 2E2OA |
| 2E2WA | 2E2ZA | 2E30B | 2E3EA | 2E3HA |
| 2E3RA | 2E3VA | 2E45A | 2E4MC | 2E4TA |
| 2E56A | 2E5FA | 2E5IA | 2E5JA | 2E5OA |
| 2E60A | 2E62A | 2E6FA | 2E6IA | 2E6JA |
| 2E6LA | 2E6OA | 2E6ZA | 2E70A | 2E72A |
| 2E7AA | 2E7CA | 2E7GA | 2E7MA | 2E7VA |
| 2E7ZA | 2E8OA | 2E8VA | 2E9XA | 2E9XB |
| 2E9XC | 2E9XD | 2EA5A | 2EA7A | 2EABA |
| 2EB6A | 2EBIA | 2EBLA | 2EBNA | 2EBVA |
| 2EC4A | 2ECCA | 2ECEA | 2ECUA | 2EDIA |
| 2EDOA | 2EDPA | 2EE7A | 2EEFA | 2EG3A |
| 2EH0A | 2EH1A | 2EHZA | 2EI9A | 2EIYA |
| 2EJ8A | 2EJEA | 2EJNA | 2EK9A | 2EKDA |
| 2EKIA | 2ELCA | 2ENDA | 2ENGA | 2ENQA |
| 2EO2A | 2EPJA | 2EPLX | 2EQ5A | 2EQGA |
| 2EQKA | 2EQNA | 2EQOA | 2EQXA | 2ERFA |
| 2ERLA | 2ERVA | 2ERWA | 2ES4D | 2ESNA |
| 2ESSA | 2ET1A | 2ETJA | 2ETSA | 2ETVA |
| 2EV1A | 2EVRA | 2EW0A | 2EWCA | 2EWFA |
| 2EWLA | 2EWTA | 2EX4A | 2EXDA | 2EXNA |
| 2EYUA | 2EZ2A | 2EZHA | 2EZKA | 2F01A |
| 2F05A | 2F1FA | 2F1KA | 2F1NA | 2F22A |
| 2F24A | 2F2HA | 2F3IA | 2F40A | 2F4IA |
| 2F4LA | 2F4MA | 2F4MB | 2F4PA | 2F4QA |
| 2F5TX | 2F5XA | 2F62A | 2F7BA | 2F7TA |
| 2F7VA | 2F96A | 2F9HA | 2F9IB | 2FA1A |
| 2FARA | 2FB0A | 2FB2A | 2FB5A | 2FB6A |
| 2FBAA | 2FCBA | 2FCJA | 2FCLA | 2FCTA |
| 2FCWA | 2FCWB | 2FDNA | 2FE0A | 2FE3A |
| 2FE9A | 2FEAA | 2FEFA | 2FELA | 2FFCA |
| 2FFKA | 2FFTA | 2FFUA | 2FFYA | 2FG1A |
| 2FGQX | 2FH1A | 2FH5A | 2FHDA | 2FHOA |
| 2FHPA | 2FHZA | 2FHZB | 2FI1A | 2FIQA |
| 2FIUA | 2FIYA | 2FJ6A | 2FJLA | 2FK9A |
| 2FKKA | 2FM8C | 2FMAA | 2FMCA | 2FN9A |
| 2FNAA | 2FNOA | 2FNUA | 2FOMA | 2FOMB |
| 2FOZA | 2FP1A | 2FP8A | 2FQHA | 2FR5A |
| 2FREA | 2FRGP | 2FS2A | 2FS5A | 2FSJA |
| 2FSQA | 2FSRA | 2FSUA | 2FTRA | 2FU2A |
| 2FU5A | 2FUFA | 2FULA | 2FUPA | 2FUQA |
| 2FURA | 2FUVA | 2FV4B | 2FVTA | 2FVYA |
| 2FWHA | 2FX5A | 2FXQA | 2FY7A | 2FYFA |
| 2FYGA | 2FYXA | 2FZ0A | 2FZPA | 2FZSA |
| 2FZTA | 2G0DA | 2G0WA | 2G1EA | 2G2CA |
| 2G2KA | 2G3BA | 2G3RA | 2G3WA | 2G40A |
| 2G50A | 2G62A | 2G64A | 2G7GA | 2G7JA |
| 2G7OA | 2G7SA | 2G80A | 2G81I | 2G8LA |
| 2G8SA | 2GA1A | 2GA8A | 2GAGC | 2GAGD |
| 2GAIA | 2GAKA | 2GB4A | 2GBOA | 2GBSA |
| 2GC7D | 2GCCA | 2GCUA | 2GD7A | 2GDMA |
| 2GDQA | 2GDTA | 2GEYA | 2GF5A | 2GFFA |
| 2GGCA | 2GGRA | 2GHFA | 2GHRA | 2GHSA |
| 2GHVC | 2GIAA | 2GIAB | 2GIBA | 2GIXA |
| 2GIYA | 2GJ3A | 2GJ4A | 2GJFA | 2GJIA |
| 2GJLA | 2GJUA | 2GJYA | 2GKEA | 2GKGA |
| 2GKPA | 2GLWA | 2GLZA | 2GMQA | 2GMYA |
| 2GNPA | 2GO9A | 2GOXB | 2GPIA | 2GPJA |
| 2GQ0A | 2GQBA | 2GQCA | 2GQKA | 2GQTA |
| 2GR8A | 2GROB | 2GS5A | 2GS8A | 2GSOA |
| 2GTRA | 2GTVX | 2GU3A | 2GUDA | 2GUFA |
| 2GUIA | 2GUTA | 2GVIA | 2GW6A | 2GWDA |
| 2GWMA | 2GWNA | 2GX5A | 2GXQA | 2GYQA |
| 2GZ4A | 2GZ6A | 2GZSA | 2GZVA | 2H1EA |
| 2H1TA | 2H1VA | 2H2MA | 2H2TB | 2H3JA |
| 2H3KA | 2H5CA | 2H5NA | 2H6FA | 2H6FB |
| 2H7AA | 2H88B | 2H88C | 2H88D | 2H8EA |
| 2H8GA | 2H98A | 2H9FA | 2HA8A | 2HAJA |
| 2HALA | 2HAYA | 2HBAA | 2HBPA | 2HC1A |
| 2HC5A | 2HCFA | 2HCMA | 2HDIB | 2HE7A |
| 2HEPA | 2HEQA | 2HEUA | 2HEWF | 2HF6A |
| 2HFDA | 2HFIA | 2HFKA | 2HFVA | 2HG7A |
| 2HGCA | 2HGKA | 2HH8A | 2HH9A | 2HHCA |
| 2HHIA | 2HHPA | 2HHZA | 2HI6A | 2HILA |
| 2HINA | 2HIYA | 2HJEA | 2HJJA | 2HJQA |
| 2HK0A | 2HKJA | 2HKVA | 2HL0A | 2HLJA |
| 2HLQA | 2HLYA | 2HMAA | 2HNGA | 2HNHA |
| 2HOXA | 2HP0A | 2HPSA | 2HPUA | 2HQ4A |
| 2HQ7A | 2HQSA | 2HQSC | 2HQYA | 2HR7A |
| 2HRKA | 2HRKB | 2HS1A | 2HS5A | 2HSBA |
| 2HSTA | 2HTAA | 2HTSA | 2HUHA | 2HV2A |
| 2HW1A | 2HW2A | 2HWTA | 2HWXA | 2HX0A |
| 2HX5A | 2HX6A | 2HY7A | 2HYTA | 2HYXA |
| 2HZCA | 2HZDA | 2HZGA | 2HZLA | 2I02A |
| 2I0KA | 2I1SA | 2I2CA | 2I2OA | 2I2XA |
| 2I2XB | 2I3OA | 2I49A | 2I4AA | 2I4IA |
| 2I51A | 2I53A | 2I54A | 2I5EA | 2I5IA |
| 2I5UA | 2I5VO | 2I68A | 2I6HA | 2I6TA |
| 2I74A | 2I7AA | 2I7DA | 2I7GA | 2I7HA |
| 2I7RA | 2I7UA | 2I8DA | 2I8LA | 2I8TA |
| 2I9CA | 2I9DA | 2I9UA | 2I9WA | 2I9XA |
| 2I9YA | 2IA1A | 2IA7A | 2IABA | 2IAFA |
| 2IAYA | 2IBGA | 2IBGE | 2IBLA | 2IC6A |
| 2ICGA | 2ICHA | 2ICUA | 2ICYA | 2ID4A |
| 2IDLA | 2IEQA | 2IF1A | 2IF6A | 2IF8A |
| 2IFOA | 2IFSA | 2IFVA | 2IFXA | 2IG8A |
| 2IGIA | 2IGSA | 2II2A | 2IIHA | 2IJ2A |
| 2IJLA | 2IJQA | 2IKDA | 2IKSA | 2ILKA |
| 2ILRA | 2IM9A | 2IMFA | 2IMHA | 2IMJA |
| 2IMQX | 2IMSA | 2IMUA | 2IMZA | 2IN3A |
| 2INUA | 2IOCA | 2IP1A | 2IP2A | 2IPBA |
| 2IQCA | 2IQTA | 2IQYA | 2IRPA | 2ISBA |
| 2IT9A | 2ITBA | 2ITHA | 2IU4A | 2IU5A |
| 2IUEA | 2IUWA | 2IUYA | 2IVFA | 2IVFC |
| 2IW1A | 2IWRA | 2IXAA | 2IXDA | 2IXSA |
| 2IXTA | 2IYVA | 2IZ3A | 2IZ6A | 2IZRA |
| 2IZXA | 2IZZA | 2J0AA | 2J0NA | 2J1VA |
| 2J3MA | 2J3XA | 2J43A | 2J48A | 2J4MA |
| 2J58A | 2J5DA | 2J5GA | 2J5HA | 2J5IA |
| 2J5YA | 2J6AA | 2J6BA | 2J6GA | 2J6LA |
| 2J73A | 2J7QA | 2J8BA | 2J8JA | 2J8KA |
| 2J8PA | 2J97A | 2J9IA | 2J9OA | 2JA4A |
| 2JA9A | 2JB7A | 2JC9A | 2JCBA | 2JCDA |
| 2JCQA | 2JDAA | 2JDCA | 2JDIA | 2JDID |
| 2JDIG | 2JDIH | 2JDII | 2JDJA | 2JE3A |
| 2JE8A | 2JEKA | 2JFRA | 2JG0A | 2JG1A |
| 2JH1A | 2JHBA | 2JHFA | 2JHNA | 2JISA |
| 2JJ7A | 2JK9A | 2JKHL | 2JKUA | 2JLIA |
| 2JLPA | 2JM2A | 2JM4A | 2JMBA | 2JMHA |
| 2JMKA | 2JMLA | 2JMPA | 2JMSA | 2JMZA |
| 2JN0A | 2JN6A | 2JN7A | 2JN9A | 2JNGA |
| 2JNSA | 2JO1A | 2JO7A | 2JOBA | 2JOEA |
| 2JOKA | 2JONA | 2JOQA | 2JOVA | 2JOXA |
| 2JOYA | 2JOZA | 2JPBA | 2JPDA | 2JPHA |
| 2JPNA | 2JPQA | 2JPUA | 2JQ3A | 2JQ5A |
| 2JQEA | 2JQNA | 2JQOA | 2JQQA | 2JQYA |
| 2JQZA | 2JR0A | 2JR1A | 2JR3A | 2JR7A |
| 2JRAA | 2JRFA | 2JRMA | 2JROA | 2JRPA |
| 2JRYA | 2JS1A | 2JS4A | 2JS5A | 2JS9A |
| 2JSNA | 2JSPA | 2JSSA | 2JSSB | 2JSWA |
| 2JSXA | 2JT1A | 2JTDA | 2JTKA | 2JTMA |
| 2JTVA | 2JTXA | 2JTYA | 2JU0B | 2JU4A |
| 2JU5A | 2JUAA | 2JUBA | 2JUGA | 2JUOA |
| 2JV2A | 2JV7A | 2JV8A | 2JVMA | 2JVUA |
| 2JVVA | 2JVWA | 2JVZA | 2JW1A | 2JWAA |
| 2JWGA | 2JWHA | 2JWYA | 2JX0A | 2JX3A |
| 2JX4A | 2JX5A | 2JX9A | 2JXCB | 2JXGA |
| 2JXNA | 2JXPA | 2JXTA | 2JY9A | 2JYEA |
| 2JYNA | 2JYSA | 2JZ1A | 2JZ3A | 2JZ4A |
| 2JZ6A | 2JZ7A | 2JZ8A | 2JZFA | 2JZLA |
| 2JZXA | 2K02A | 2K03B | 2K0AA | 2K0GA |
| 2K0MA | 2K0NA | 2K0QA | 2K13X | 2K14A |
| 2K19A | 2K1OA | 2K21A | 2K27A | 2K29A |
| 2K2BA | 2K2DA | 2K2EA | 2K2OA | 2K2SB |
| 2K2VA | 2K2WA | 2K31A | 2K37A | 2K3AA |
| 2K3DA | 2K3FA | 2K3IA | 2K3JA | 2K3NA |
| 2K3OA | 2K3PA | 2K3RA | 2K46A | 2K47A |
| 2K4FA | 2K4NA | 2K4QA | 2K4TA | 2K4VA |
| 2K4XA | 2K4YA | 2K4ZA | 2K50A | 2K53A |
| 2K5CA | 2K5DA | 2K5IA | 2K5JA | 2K5KA |
| 2K5PA | 2K5RA | 2K5SA | 2K5TA | 2K5UA |
| 2K5VA | 2K60A | 2K6GA | 2K6IA | 2K6LA |
| 2K6XA | 2K73A | 2K75A | 2K7MA | 2K7QA |
| 2K7RA | 2K85A | 2K87A | 2K88A | 2K89A |
| 2K8EA | 2K8OA | 2K8PA | 2K8QA | 2K8SA |
| 2K8YA | 2K9AA | 2K9HA | 2K9MA | 2K9PA |
| 2K9SA | 2K9YA | 2KA4B | 2KA6B | 2KAKA |
| 2KATA | 2KB9A | 2KBBA | 2KBGA | 2KBIA |
| 2KBNA | 2KBQA | 2KBZA | 2KC2A | 2KC5A |
| 2KC6A | 2KCAA | 2KCDA | 2KCNA | 2KCOA |
| 2KCQA | 2KCTA | 2KCZA | 2KD1A | 2KD2A |
| 2KD3A | 2KDDA | 2KDOA | 2KDPA | 2KDTA |
| 2KDXA | 2KE4A | 2KELA | 2KESA | 2KEYA |
| 2KFDA | 2KFSA | 2KFVA | 2KG4A | 2KGJA |
| 2KHKA | 2KHMA | 2KHQA | 2KHVA | 2KI7A |
| 2KICA | 2KIEA | 2KIFA | 2KIGA | 2KIIA |
| 2KIJA | 2KINA | 2KINB | 2KISA | 2KIVA |
| 2KIWA | 2KJ1A | 2KJ3A | 2KJEB | 2KJFA |
| 2KJGA | 2KJIA | 2KJQA | 2KJXA | 2KJYA |
| 2KK1A | 2KK2A | 2KK4A | 2KK7A | 2KK9A |
| 2KKEA | 2KKHA | 2KKMA | 2KKNA | 2KKPA |
| 2KKSA | 2KKTA | 2KKVA | 2KKXA | 2KL5A |
| 2KL8A | 2KLAA | 2KLBA | 2KLDA | 2KLLA |
| 2KLOA | 2KLQA | 2KLUA | 2KLZA | 2KM1A |
| 2KM4A | 2KMAA | 2KMB1 | 2KMGA | 2KMLA |
| 2KMMA | 2KMUA | 2KMWA | 2KN0A | 2KN8A |
| 2KNCA | 2KNCB | 2KNGA | 2KNIA | 2KNJA |
| 2KNQA | 2KO2A | 2KO6A | 2KOBA | 2KOEA |
| 2KONA | 2KOUA | 2KOYA | 2KP6A | 2KPJA |
| 2KPMA | 2KPQA | 2KPTA | 2KPUA | 2KQ1A |
| 2KQ2A | 2KQ5A | 2KQ9A | 2KQRA | 2KQVA |
| 2KR0A | 2KR6A | 2KR7A | 2KRCA | 2KREA |
| 2KRTA | 2KRUA | 2KRXA | 2KSDA | 2KSEA |
| 2KSFA | 2KSGA | 2KSKA | 2KSLA | 2KSNA |
| 2KSWA | 2KT5B | 2KT7A | 2KT8A | 2KT9A |
| 2KTAA | 2KTEA | 2KTLA | 2KTRA | 2KTUA |
| 2KUAA | 2KUBA | 2KUIA | 2KUMA | 2KUOA |
| 2KUTA | 2KUYA | 2KVAA | 2KVLA | 2KVPA |
| 2KVRA | 2KVSA | 2KVTA | 2KVUA | 2KVVA |
| 2KW0A | 2KW3A | 2KW3C | 2KW5A | 2KW7A |
| 2KWAA | 2KWHA | 2KWPA | 2KWQA | 2KWUA |
| 2KWVA | 2KWYA | 2KWZA | 2KX2A | 2KX7A |
| 2KXCB | 2KXEA | 2KXFA | 2KXGA | 2KXIA |
| 2KXOA | 2KXSA | 2KXTA | 2KXYA | 2KY5A |
| 2KYHA | 2KYWA | 2KYYA | 2KYZA | 2KZ3A |
| 2KZ4A | 2KZ5A | 2KZ6A | 2KZ7C | 2KZ9A |
| 2KZBA | 2KZHA | 2KZRA | 2KZSA | 2KZTA |
| 2KZXA | 2L02A | 2L03A | 2L04A | 2L08A |
| 2L0CA | 2L0JA | 2L0KA | 2L0ZA | 2L10A |
| 2L16A | 2L1AA | 2L1CA | 2L1IA | 2L1LB |
| 2L1NA | 2L1QA | 2L1SA | 2L1TA | 2L21A |
| 2L23A | 2L25A | 2L2DA | 2L2IB | 2L2LA |
| 2L2OA | 2L32A | 2L35A | 2L37A | 2L3LA |
| 2L3NA | 2L3OA | 2L3RA | 2L3UA | 2L3YA |
| 2L42A | 2L48A | 2L4AA | 2L4ZA | 2L5AA |
| 2L5CA | 2L5FA | 2L5GB | 2L5QA | 2L5VA |
| 2L60A | 2L69A | 2L6AA | 2L6FA | 2L6OA |
| 2L74A | 2L76A | 2L7KA | 2L7PA | 2L7SA |
| 2L7XA | 2L7YA | 2L81A | 2L82A | 2L8DA |
| 2L8EA | 2L8KA | 2L8LA | 2L8NA | 2L8OA |
| 2L8TA | 2L93A | 2L9BA | 2L9BB | 2L9DA |
| 2L9FA | 2L9JA | 2L9MA | 2L9QA | 2L9SA |
| 2L9UA | 2L9WA | 2LA3A | 2LA4A | 2LA7A |
| 2LAHA | 2LAIA | 2LBOA | 2LC0A | 2LCKA |
| 2LCLA | 2LCQA | 2LCRA | 2LCUA | 2LCYA |
| 2LD3A | 2LD7A | 2LD7B | 2LEHA | 2LEQA |
| 2LEZA | 2LF0A | 2LFCA | 2LFEA | 2LFPA |
| 2LFUA | 2LFVA | 2LFWB | 2LG1A | 2LG4A |
| 2LG7A | 2LGNA | 2LGVA | 2LGXA | 2LGYA |
| 2LH0A | 2LHFA | 2LHKA | 2LHNA | 2LHRA |
| 2LHTA | 2LIEA | 2LIOA | 2LISA | 2LIYA |
| 2LJ9A | 2LJUA | 2LJWA | 2LK4A | 2LKGA |
| 2LKLA | 2LKMB | 2LKNA | 2LKOA | 2LL0A |
| 2LLGA | 2LLHA | 2LLIA | 2LLLA | 2LLVA |
| 2LLXA | 2LLZA | 2LM0A | 2LM4A | 2LMDA |
| 2LMKA | 2LMLA | 2LMZA | 2LN3A | 2LN7A |
| 2LN8A | 2LNAA | 2LNBA | 2LNJA | 2LNLA |
| 2LNMA | 2LNSA | 2LNVA | 2LO0A | 2LO4A |
| 2LOEA | 2LONA | 2LOQA | 2LORA | 2LOSA |
| 2LOTA | 2LOXA | 2LOXB | 2LPBA | 2LPEA |
| 2LPNA | 2LPUA | 2LPYA | 2LQ4p | 2LQ6A |
| 2LQ7A | 2LQGA | 2LQHB | 2LQJA | 2LQLA |
| 2LQTA | 2LQUA | 2LQVA | 2LQXA | 2LR4A |
| 2LRAA | 2LRDA | 2LRIC | 2LRJA | 2LRMA |
| 2LS01 | 2LSEA | 2LSHA | 2LSJA | 2LSLA |
| 2LSMA | 2LSWA | 2LT1A | 2LT2A | 2LT3A |
| 2LT8A | 2LTFA | 2LTLA | 2LTNB | 2LTRA |
| 2LTSA | 2LU2A | 2LUAA | 2LUHA | 2LUHB |
| 2LUUA | 2LUWA | 2LUYA | 2LV4A | 2LV5A |
| 2LVGA | 2LVHA | 2LVLA | 2LVSA | 2LVWA |
| 2LVXA | 2LW1A | 2LW3A | 2LW6A | 2LW7A |
| 2LW9A | 2LWDA | 2LWFA | 2LWLA | 2LWWB |
| 2LWYA | 2LXEA | 2LXLA | 2LXRA | 2LXWA |
| 2LY1A | 2LY4B | 2LYCA | 2LYDA | 2LYHA |
| 2LYIA | 2LYXA | 2LYYA | 2LZ0A | 2LZFA |
| 2LZJA | 2LZLA | 2LZNA | 2M0AA | 2M0GA |
| 2M0OA | 2M13A | 2M14B | 2M1CA | 2M1HA |
| 2M1LA | 2M1MA | 2M1SA | 2M20A | 2M25A |
| 2M26A | 2M2BA | 2M2FA | 2M2KA | 2M3DA |
| 2M3KA | 2M3LA | 2M47A | 2M48A | 2M4GA |
| 2M4HA | 2M4LA | 2M4NA | 2M4VA | 2M5OA |
| 2M5ZA | 2M67A | 2M6BA | 2M71A | 2M74A |
| 2M76A | 2M7AA | 2M7NA | 2M7OA | 2M7QA |
| 2M7YA | 2M85A | 2M86A | 2M89A | 2M8EA |
| 2M8LA | 2M8PA | 2M9MA | 2M9UA | 2M9VA |
| 2M9WA | 2MA6A | 2MB9A | 2MBHB | 2MBKA |
| 2MBLA | 2MBYA | 2MC2A | 2MC3A | 2MC4A |
| 2MCAA | 2MCDA | 2MCMA | 2MCQA | 2MCTA |
| 2MDGA | 2MDTA | 2MH4A | 2MHDA | 2MHRA |
| 2MPRA | 2MUCA | 2NL9A | 2NLRA | 2NLVA |
| 2NMLA | 2NOCA | 2NOOA | 2NPBA | 2NPNA |
| 2NQRA | 2NQTA | 2NQWA | 2NR5A | 2NR7A |
| 2NRGA | 2NRKA | 2NRRA | 2NS0A | 2NSCA |
| 2NSZA | 2NT0A | 2NTEA | 2NUHA | 2NUJA |
| 2NW0A | 2NW2A | 2NW8A | 2NWFA | 2NWHA |
| 2NWTA | 2NX2A | 2NXFA | 2NXUA | 2NXVA |
| 2NXWA | 2NYIA | 2NZXA | 2O07A | 2O0MA |
| 2O0YA | 2O14A | 2O16A | 2O1KA | 2O1QA |
| 2O1SA | 2O2VA | 2O2XA | 2O3IA | 2O3LA |
| 2O4VA | 2O5UA | 2O5VA | 2O62A | 2O6PA |
| 2O6SA | 2O6XA | 2O70A | 2O7AA | 2O7IA |
| 2O7MA | 2O7RA | 2O8GI | 2O8PA | 2O90A |
| 2O9SA | 2OA2A | 2OA4A | 2OA9A | 2OAFA |
| 2OB3A | 2OB5A | 2OBPA | 2OCGA | 2OCTA |
| 2OD4A | 2OD5A | 2OD6A | 2ODAA | 2ODFA |
| 2ODKA | 2OEBA | 2OF3A | 2OFCA | 2OFKA |
| 2OFZA | 2OGFA | 2OGGA | 2OH1A | 2OH3A |
| 2OH5A | 2OIKA | 2OITA | 2OIWA | 2OIZA |
| 2OIZD | 2OJ6A | 2OJHA | 2OJLA | 2OJWA |
| 2OK5A | 2OKFA | 2OKGA | 2OKMA | 2OKTA |
| 2OKUA | 2OLBA | 2OLMA | 2OLNA | 2OLRA |
| 2OMDA | 2OMLA | 2OOBA | 2OOKA | 2OPCA |
| 2OPLA | 2OQGA | 2OQZA | 2ORWA | 2OSOA |
| 2OSVA | 2OSXA | 2OT2A | 2OT3A | 2OTRA |
| 2OU1A | 2OU3A | 2OU6A | 2OUIA | 2OUTA |
| 2OV0A | 2OV6A | 2OVGA | 2OVJA | 2OWAA |
| 2OX7A | 2OXGA | 2OXGB | 2OY7A | 2OYAA |
| 2OYCA | 2OYOA | 2OZ8A | 2OZEA | 2OZGA |
| 2OZHA | 2OZJA | 2OZLA | 2OZTA | 2P02A |
| 2P09A | 2P0AA | 2P0BA | 2P0MA | 2P0NA |
| 2P0OA | 2P0SA | 2P0WA | 2P10A | 2P12A |
| 2P14A | 2P17A | 2P19A | 2P1MA | 2P1MB |
| 2P2DA | 2P2SA | 2P3EA | 2P3PA | 2P45B |
| 2P4FA | 2P4GA | 2P4HX | 2P4OA | 2P4SA |
| 2P51A | 2P58A | 2P58B | 2P58C | 2P5KA |
| 2P6PA | 2P6WA | 2P8GA | 2P8IA | 2P8JA |
| 2P97A | 2P9HA | 2P9WA | 2PA7A | 2PBDV |
| 2PBIA | 2PBKA | 2PD1A | 2PD2A | 2PF5A |
| 2PFIA | 2PFUA | 2PFZA | 2PG3A | 2PGNA |
| 2PHNA | 2PIEA | 2PIMA | 2PJHA | 2PKEA |
| 2PKFA | 2PMAA | 2PN0A | 2PNDA | 2PNEA |
| 2PNLA | 2PNWA | 2POCA | 2POHA | 2PORA |
| 2PP4A | 2PPQA | 2PPVA | 2PPXA | 2PQ7A |
| 2PQXA | 2PR7A | 2PRVA | 2PS1A | 2PSPA |
| 2PSTX | 2PTMA | 2PTRA | 2PU3A | 2PUZA |
| 2PVBA | 2PW8I | 2PW9A | 2PX9A | 2PXGA |
| 2PXRC | 2PYQA | 2PYXA | 2Q01A | 2Q02A |
| 2Q03A | 2Q0SA | 2Q0TA | 2Q22A | 2Q28A |
| 2Q2RA | 2Q35A | 2Q3MA | 2Q3QA | 2Q3SA |
| 2Q3TA | 2Q40A | 2Q43A | 2Q48A | 2Q4BA |
| 2Q4FA | 2Q4KA | 2Q4MA | 2Q4NA | 2Q4OA |
| 2Q4PA | 2Q4XA | 2Q4ZA | 2Q5CA | 2Q62A |
| 2Q78A | 2Q7AA | 2Q7WA | 2Q83A | 2Q8KA |
| 2Q8UA | 2Q99A | 2Q9RA | 2Q9UA | 2QAPA |
| 2QB7A | 2QCPX | 2QDLA | 2QDXA | 2QE8A |
| 2QEBA | 2QEEA | 2QEUA | 2QF4A | 2QFAB |
| 2QFAC | 2QFEA | 2QG6A | 2QG8A | 2QGUA |
| 2QGYA | 2QHFA | 2QHQA | 2QIBA | 2QIPA |
| 2QISA | 2QJFA | 2QJLA | 2QJTA | 2QJVA |
| 2QJWA | 2QJZA | 2QK1A | 2QK7B | 2QKHA |
| 2QKHB | 2QKPA | 2QL3A | 2QL8A | 2QLXA |
| 2QM4A | 2QM8A | 2QMCA | 2QMCB | 2QMLA |
| 2QNEA | 2QNGA | 2QNKA | 2QNLA | 2QNUA |
| 2QOLA | 2QP2A | 2QPWA | 2QPXA | 2QQ8A |
| 2QQIA | 2QQZA | 2QR4A | 2QRUA | 2QRYA |
| 2QSAA | 2QSBA | 2QSIA | 2QSKA | 2QSQA |
| 2QSWA | 2QTXA | 2QUDA | 2QUOA | 2QV3A |
| 2QV5A | 2QV8A | 2QVKA | 2QW5A | 2QWOB |
| 2QWUA | 2QXFA | 2QXSA | 2QYCA | 2QYFA |
| 2QYFB | 2QYZA | 2QZCA | 2QZQA | 2QZUA |
| 2R01A | 2R0XA | 2R10A | 2R11A | 2R16A |
| 2R1IA | 2R2AA | 2R2CA | 2R2ZA | 2R31A |
| 2R3SA | 2R46A | 2R47A | 2R4IA | 2R4QA |
| 2R4TA | 2R51A | 2R5OA | 2R5XA | 2R6UA |
| 2R751 | 2R7GA | 2R85A | 2R8EA | 2R8RA |
| 2R91A | 2R9FA | 2R9QA | 2RA1A | 2RA9A |
| 2RASA | 2RAUA | 2RBCA | 2RBDA | 2RBGA |
| 2RBKA | 2RC3A | 2RCCA | 2RCIA | 2RCKA |
| 2RDCA | 2RDEA | 2RDGA | 2RDQA | 2REEA |
| 2RFFA | 2RFQA | 2RFRA | 2RGQA | 2RH0A |
| 2RH2A | 2RH3A | 2RHFA | 2RHSA | 2RHSB |
| 2RI7A | 2RIHA | 2RIJA | 2RIKA | 2RILA |
| 2RINA | 2RIQA | 2RIVB | 2RJ2A | 2RJZA |
| 2RK3A | 2RKNA | 2RKQA | 2RKVA | 2RLDA |
| 2RLOA | 2RMSB | 2RN7A | 2RNGA | 2RNRB |
| 2RO1A | 2ROHA | 2ROOA | 2ROPA | 2ROQA |
| 2ROVA | 2RP4A | 2RPAA | 2RPCA | 2RPQB |
| 2RPRA | 2RQPA | 2RQXA | 2RQYA | 2RR3B |
| 2RR9C | 2RRDA | 2RREA | 2RRFA | 2RRLA |
| 2RRNA | 2RSXA | 2RT5A | 2RT6A | 2SAKA |
| 2SICI | 2SQCA | 2TGIA | 2TPSA | 2TRCP |
| 2UTGA | 2UU8A | 2UURA | 2UUUA | 2UUYB |
| 2UV4A | 2UVKA | 2UVOA | 2UVPA | 2UW1A |
| 2UWAA | 2UWQA | 2UX0A | 2UX9A | 2UXQA |
| 2UYQA | 2UYTA | 2UYZA | 2UYZB | 2UZ1A |
| 2V03A | 2V0UA | 2V1MA | 2V1NA | 2V1QA |
| 2V25A | 2V2GA | 2V33A | 2V3GA | 2V3IA |
| 2V3SA | 2V3ZA | 2V4VA | 2V4XA | 2V5EB |
| 2V5MA | 2V6GA | 2V6KA | 2V6XA | 2V6XB |
| 2V6ZM | 2V75A | 2V76A | 2V78A | 2V79A |
| 2V7FA | 2V7KA | 2V7QJ | 2V89A | 2V8FA |
| 2V8KA | 2V8TA | 2V9HA | 2V9KA | 2V9LA |
| 2V9VA | 2VACA | 2VB1A | 2VBKA | 2VBUA |
| 2VC8A | 2VCHA | 2VCLA | 2VDAA | 2VE8A |
| 2VECA | 2VEFA | 2VEZA | 2VFKA | 2VFOA |
| 2VFRA | 2VGAA | 2VGOC | 2VGXA | 2VH4A |
| 2VHAA | 2VHKA | 2VK2A | 2VK8A | 2VKIA |
| 2VKLA | 2VKSA | 2VLGA | 2VLIA | 2VLQA |
| 2VLQB | 2VM6A | 2VN6A | 2VN6B | 2VNGA |
| 2VOBA | 2VOVA | 2VOYI | 2VPAA | 2VPBA |
| 2VPTA | 2VQ2A | 2VQ3A | 2VQCA | 2VQGA |
| 2VQPA | 2VRDA | 2VRSA | 2VRYA | 2VS0A |
| 2VSEA | 2VSMB | 2VTWA | 2VTYA | 2VUWA |
| 2VVEA | 2VVPA | 2VVWA | 2VW8A | 2VWSA |
| 2VXBA | 2VXEA | 2VXNA | 2VXTI | 2VXXA |
| 2VXZA | 2VY8A | 2VZBA | 2VZCA | 2VZPA |
| 2VZYA | 2W0BA | 2W0IA | 2W0NA | 2W0TA |
| 2W15A | 2W1JA | 2W1SA | 2W1VA | 2W2KA |
| 2W2RA | 2W31A | 2W39A | 2W3GA | 2W3PA |
| 2W3QA | 2W3XA | 2W40A | 2W491 | 2W492 |
| 2W4SA | 2W5QA | 2W61A | 2W6AA | 2W6DA |
| 2W72A | 2W7AA | 2W7ZA | 2W86A | 2W8TA |
| 2W8XA | 2W91A | 2W9OA | 2W9YA | 2WAAA |
| 2WAGA | 2WANA | 2WAOA | 2WAWA | 2WAXB |
| 2WB0X | 2WB3A | 2WBFX | 2WBMA | 2WBRA |
| 2WCRA | 2WCWA | 2WCYA | 2WD6A | 2WDCA |
| 2WDQC | 2WDQD | 2WDSA | 2WF7A | 2WFBA |
| 2WFIA | 2WFOA | 2WFPA | 2WFSA | 2WG7A |
| 2WGKA | 2WGNB | 2WGOA | 2WH6A | 2WHLA |
| 2WHMA | 2WI8A | 2WIYA | 2WJ5A | 2WJ9A |
| 2WJEA | 2WJNC | 2WJNH | 2WJNL | 2WJNM |
| 2WJRA | 2WK1A | 2WKKA | 2WKQA | 2WL1A |
| 2WLRA | 2WLUA | 2WLVA | 2WMFA | 2WN3A |
| 2WN9A | 2WNFA | 2WNMA | 2WNPF | 2WNVB |
| 2WNYA | 2WO3B | 2WOEA | 2WOJA | 2WOLA |
| 2WOYA | 2WPVB | 2WPXA | 2WQ4A | 2WQFA |
| 2WQGA | 2WQKA | 2WQRA | 2WR8A | 2WSDA |
| 2WTEA | 2WTGA | 2WTMA | 2WTPA | 2WTXA |
| 2WUJA | 2WUQA | 2WURA | 2WUUA | 2WUXA |
| 2WVWI | 2WVXA | 2WW5A | 2WW8A | 2WXUA |
| 2WY3A | 2WY3B | 2WY4A | 2WY8A | 2WY8Q |
| 2WYAA | 2WYHA | 2WZ1A | 2WZ8A | 2WZBA |
| 2WZKA | 2WZOA | 2X0DA | 2X0QA | 2X1DA |
| 2X27X | 2X2EA | 2X2HA | 2X2SA | 2X2UA |
| 2X2VA | 2X31A | 2X31G | 2X32A | 2X3CA |
| 2X3GA | 2X3HA | 2X3JA | 2X3MA | 2X43S |
| 2X46A | 2X49A | 2X4JA | 2X4KA | 2X4LA |
| 2X4WA | 2X55A | 2X5CA | 2X5FA | 2X5NA |
| 2X5OA | 2X5PA | 2X5QA | 2X5XA | 2X5YA |
| 2X61A | 2X65A | 2X6WA | 2X7JA | 2X8NA |
| 2X8RA | 2X9OA | 2X9ZA | 2XA6A | 2XAPA |
| 2XAUA | 2XBGA | 2XCMC | 2XCME | 2XDGA |
| 2XDHA | 2XDWA | 2XESA | 2XETA | 2XEUA |
| 2XF3A | 2XF7A | 2XFGB | 2XFRA | 2XFVA |
| 2XG5B | 2XGFA | 2XGRA | 2XHAA | 2XHFA |
| 2XHGA | 2XI7A | 2XIGA | 2XIJA | 2XIOA |
| 2XIWA | 2XJ4A | 2XJPA | 2XKIA | 2XLGA |
| 2XLTA | 2XM5A | 2XMIA | 2XMJA | 2XN7A |
| 2XNQA | 2XOCA | 2XODA | 2XOLA | 2XOMA |
| 2XOVA | 2XPPA | 2XPPB | 2XPWA | 2XQQA |
| 2XQUA | 2XRHA | 2XRWA | 2XRYA | 2XSEA |
| 2XSGA | 2XSUA | 2XT2A | 2XTLA | 2XTPA |
| 2XTSA | 2XU0A | 2XU2A | 2XU3A | 2XU8A |
| 2XUVA | 2XV9A | 2XVMA | 2XVSA | 2XVTA |
| 2XVYA | 2XW6A | 2XWPA | 2XWSA | 2XWTC |
| 2XWVA | 2XXNA | 2XXPA | 2XYKA | 2XZ8A |
| 2XZ9A | 2XZBB | 2XZEA | 2XZEQ | 2XZIA |
| 2Y0OA | 2Y1BA | 2Y1EA | 2Y1QA | 2Y24A |
| 2Y27A | 2Y28A | 2Y2MA | 2Y31A | 2Y39A |
| 2Y3CA | 2Y3QA | 2Y4RA | 2Y4YA | 2Y4ZA |
| 2Y53A | 2Y5PA | 2Y6HA | 2Y6XA | 2Y71A |
| 2Y78A | 2Y7CA | 2Y7CB | 2Y7EA | 2Y7LA |
| 2Y7PA | 2Y8GA | 2Y8KA | 2Y8NA | 2Y8NB |
| 2Y9UA | 2Y9WC | 2YAVA | 2YB1A | 2YBB1 |
| 2YBB2 | 2YBB4 | 2YBB5 | 2YBB6 | 2YBB7 |
| 2YBBB | 2YBBC | 2YBBD | 2YBBF | 2YBBG |
| 2YBBH | 2YBBI | 2YBBJ | 2YBBK | 2YBYA |
| 2YC3A | 2YCIX | 2YCLA | 2YD6A | 2YEQA |
| 2YEVC | 2YEWB | 2YEWC | 2YFDA | 2YFOA |
| 2YFRA | 2YFUA | 2YG2A | 2YG9A | 2YGDA |
| 2YGGA | 2YGOA | 2YH5A | 2YH6A | 2YHAA |
| 2YHCA | 2YHGA | 2YHSA | 2YI9A | 2YICA |
| 2YILA | 2YIMA | 2YJGA | 2YK4A | 2YKFA |
| 2YKZA | 2YLBA | 2YLNA | 2YMMA | 2YMVA |
| 2YMYA | 2YN0A | 2YN7A | 2YNAA | 2YNQA |
| 2YNYA | 2YOGA | 2YOPA | 2YORA | 2YPVA |
| 2YQPA | 2YRBA | 2YRCA | 2YREA | 2YRGA |
| 2YRKA | 2YRMA | 2YRNA | 2YRPA | 2YRQA |
| 2YRUA | 2YRVA | 2YS0A | 2YS9A | 2YSZA |
| 2YU3A | 2YU4A | 2YU6A | 2YUDA | 2YUEA |
| 2YUFA | 2YUGA | 2YUHA | 2YUKA | 2YUSA |
| 2YV0X | 2YVIA | 2YVPA | 2YVRA | 2YVTA |
| 2YWLA | 2YX0A | 2YXNA | 2YYKA | 2YYSA |
| 2YYYA | 2YZ0A | 2YZCA | 2Z07A | 2Z0AA |
| 2Z0DA | 2Z0JA | 2Z0TA | 2Z0XA | 2Z14A |
| 2Z15A | 2Z1DA | 2Z26A | 2Z2NA | 2Z30B |
| 2Z43A | 2Z4DA | 2Z51A | 2Z59A | 2Z5EA |
| 2Z5WA | 2Z6OA | 2Z6RA | 2Z72A | 2Z7BA |
| 2Z84A | 2Z8FA | 2Z8XA | 2Z98A | 2Z9WA |
| 2ZA4B | 2ZB4A | 2ZBCA | 2ZBLA | 2ZCAA |
| 2ZCMA | 2ZDPA | 2ZDSA | 2ZE7A | 2ZEXA |
| 2ZF9A | 2ZFDB | 2ZFGA | 2ZFIA | 2ZFYA |
| 2ZFZA | 2ZGWA | 2ZHJA | 2ZK9X | 2ZKMX |
| 2ZL7A | 2ZNRA | 2ZPMA | 2ZPTX | 2ZQ0A |
| 2ZQ5A | 2ZQEA | 2ZQMA | 2ZQOA | 2ZS0D |
| 2ZSIB | 2ZTBA | 2ZUVA | 2ZUXA | 2ZWAA |
| 2ZWSA | 2ZX0A | 2ZXQA | 2ZYLA | 2ZYRA |
| 2ZZ8A | 2ZZ9A | 2ZZJA | 2ZZVA | 3A02A |
| 3A07A | 3A09A | 3A0OA | 3A0ZA | 3A15A |
| 3A1DA | 3A1PB | 3A2ZA | 3A35A | 3A38A |
| 3A3DA | 3A54A | 3A57A | 3A5FA | 3A5PA |
| 3A5YA | 3A6RA | 3A72A | 3A77A | 3A8GA |
| 3A8GB | 3A8TA | 3A9FA | 3A9IA | 3A9SA |
| 3AA0A | 3AA0B | 3AALA | 3ABDA | 3ABDX |
| 3ACHA | 3ACXA | 3AEHA | 3AEKA | 3AEKB |
| 3AFOA | 3AG3A | 3AG3B | 3AG3C | 3AG3D |
| 3AG3E | 3AG3F | 3AG3G | 3AG3H | 3AG3I |
| 3AG3J | 3AG3K | 3AG3L | 3AG3M | 3AGNA |
| 3AHCA | 3AHNA | 3AHYA | 3AIAA | 3AIIA |
| 3AJ4A | 3AJ6A | 3AJ7A | 3AJDA | 3AJIB |
| 3AJRA | 3AK8A | 3AKJA | 3AKOB | 3AKSA |
| 3AL2A | 3ALJA | 3ALRA | 3AMIA | 3AMLA |
| 3AMNA | 3AMRA | 3ANOA | 3AOFA | 3AONA |
| 3AONB | 3AOWA | 3AP9A | 3APOA | 3APQA |
| 3AQ2A | 3AS8A | 3ASIA | 3ASLA | 3ATSA |
| 3ATVA | 3AVRA | 3AWMA | 3AWUA | 3AWUB |
| 3AX2A | 3AXBA | 3AY2A | 3AYHA | 3AYHB |
| 3AZOA | 3B02A | 3B08B | 3B0FA | 3B0GA |
| 3B0XA | 3B1BA | 3B21A | 3B2YA | 3B33A |
| 3B34A | 3B3FA | 3B40A | 3B49A | 3B4NA |
| 3B4QA | 3B4UA | 3B55A | 3B5EA | 3B5MA |
| 3B5OA | 3B5QA | 3B64A | 3B6EA | 3B79A |
| 3B7CA | 3B7HA | 3B8FA | 3B9TA | 3B9WA |
| 3BA3A | 3BALA | 3BB0A | 3BB7A | 3BBJA |
| 3BBUA | 3BBZA | 3BC8A | 3BC9A | 3BCHA |
| 3BCWA | 3BCYA | 3BCZA | 3BDIA | 3BDVA |
| 3BE6A | 3BEDA | 3BEEA | 3BEIA | 3BEMA |
| 3BEXA | 3BFMA | 3BFOA | 3BFQG | 3BGEA |
| 3BGYA | 3BHDA | 3BHYA | 3BI7A | 3BIQA |
| 3BIXA | 3BIYA | 3BIZA | 3BJ5A | 3BJDA |
| 3BJEA | 3BJNA | 3BJQA | 3BJVA | 3BK5A |
| 3BL9A | 3BLNA | 3BLZA | 3BM1A | 3BMZA |
| 3BN3B | 3BNJA | 3BO6A | 3BODA | 3BOEA |
| 3BOFA | 3BOGA | 3BONA | 3BOSA | 3BPKA |
| 3BPTA | 3BQ3A | 3BQAA | 3BQPA | 3BQWA |
| 3BRCA | 3BS4A | 3BS6A | 3BT5A | 3BUUA |
| 3BV8A | 3BVFA | 3BVXA | 3BWHA | 3BWLA |
| 3BWSA | 3BWUD | 3BWUF | 3BWWA | 3BWZA |
| 3BXPA | 3BY9A | 3BYPA | 3BYQA | 3BZ6A |
| 3C02A | 3C0NA | 3C18A | 3C1MA | 3C1QA |
| 3C2QA | 3C2UA | 3C3DA | 3C3VA | 3C48A |
| 3C4BA | 3C4SA | 3C57A | 3C5KA | 3C5NA |
| 3C6AA | 3C6CA | 3C70A | 3C7FA | 3C7JA |
| 3C7XA | 3C85A | 3C8CA | 3C8DA | 3C8LA |
| 3C8WA | 3C8YA | 3C9AA | 3C9AC | 3C9FA |
| 3C9HA | 3C9PA | 3C9QA | 3C9ZA | 3CA8A |
| 3CANA | 3CAXA | 3CBWA | 3CBZA | 3CCDA |
| 3CDUA | 3CE9A | 3CEBA | 3CECA | 3CEUA |
| 3CEXA | 3CG3A | 3CGXA | 3CH0A | 3CHBD |
| 3CHJA | 3CHMA | 3CI0I | 3CI0K | 3CI3A |
| 3CI6A | 3CI9A | 3CIJA | 3CIMA | 3CINA |
| 3CIPG | 3CITA | 3CJEA | 3CJLA | 3CJMA |
| 3CJNA | 3CJPA | 3CJSA | 3CJSB | 3CJWA |
| 3CJYA | 3CK1A | 3CK6A | 3CKCA | 3CKJA |
| 3CKKA | 3CKMA | 3CL5A | 3CLAA | 3CLJA |
| 3CLMA | 3CLQA | 3CLSC | 3CLSD | 3CM3A |
| 3CMGA | 3CNEA | 3CNRA | 3CNUA | 3CNVA |
| 3CNYA | 3COVA | 3CP0A | 3CP3A | 3CP7A |
| 3CPOA | 3CQ0A | 3CQBA | 3CQLA | 3CQYA |
| 3CRDA | 3CRYA | 3CSVA | 3CT1A | 3CT6A |
| 3CT9A | 3CTPA | 3CTVA | 3CTZA | 3CU2A |
| 3CU9A | 3CUZA | 3CV0A | 3CVJA | 3CVOA |
| 3CW8X | 3CWCA | 3CWFA | 3CWWA | 3CXKA |
| 3CXNA | 3CYPB | 3CZ1A | 3CZ7A | 3CZ8A |
| 3CZPA | 3CZXA | 3D01A | 3D02A | 3D03A |
| 3D06A | 3D0FA | 3D0JA | 3D0KA | 3D1CA |
| 3D1LA | 3D1PA | 3D1RA | 3D2OA | 3D2QA |
| 3D30A | 3D32A | 3D33A | 3D34A | 3D3BA |
| 3D3BJ | 3D3OA | 3D3YA | 3D40A | 3D4EA |
| 3D4UB | 3D55A | 3D5PA | 3D6WA | 3D7IA |
| 3D7RA | 3D85C | 3D85D | 3D89A | 3D8PA |
| 3D9AH | 3D9NA | 3D9XA | 3DA0A | 3DA8A |
| 3DALA | 3DB2A | 3DB7A | 3DCDA | 3DCLA |
| 3DCPA | 3DCZA | 3DD7A | 3DDCB | 3DEFA |
| 3DELB | 3DF6A | 3DF8A | 3DFFA | 3DFGA |
| 3DFUA | 3DGPA | 3DGPB | 3DHAA | 3DHXA |
| 3DJEA | 3DJLA | 3DK9A | 3DKMA | 3DKRA |
| 3DLCA | 3DLOA | 3DLQR | 3DM8A | 3DMCA |
| 3DMGA | 3DMLA | 3DMNA | 3DN7A | 3DNFA |
| 3DNHA | 3DNJA | 3DNLB | 3DNSA | 3DNUA |
| 3DNXA | 3DO6A | 3DO8A | 3DOUA | 3DP7A |
| 3DQPA | 3DQYA | 3DRFA | 3DS2A | 3DS8A |
| 3DSBA | 3DSKA | 3DSMA | 3DSSA | 3DT5A |
| 3DTZA | 3DWAA | 3DWCA | 3DX5A | 3DXLA |
| 3DXTA | 3DXYA | 3DZAA | 3E03A | 3E08A |
| 3E0RA | 3E0XA | 3E0ZA | 3E11A | 3E15A |
| 3E18A | 3E2OA | 3E35A | 3E38A | 3E3MA |
| 3E48A | 3E4GA | 3E4VA | 3E4WA | 3E50C |
| 3E56A | 3E59A | 3E5TA | 3E5XA | 3E7AA |
| 3E7HA | 3E7KA | 3E7NA | 3E7UX | 3E8LC |
| 3E8OA | 3E8TA | 3E9KA | 3E9SA | 3EA0A |
| 3EA1A | 3EA6A | 3EAFA | 3EATX | 3EBBA |
| 3EBTA | 3EC3A | 3EC4A | 3ECFA | 3ECHA |
| 3EDFA | 3EDOA | 3EDVA | 3EDYA | 3EE4A |
| 3EE9A | 3EEHA | 3EERA | 3EF8A | 3EFEA |
| 3EG4A | 3EGGC | 3EGLA | 3EGWC | 3EH1A |
| 3EHGA | 3EIKA | 3EJKA | 3EJXA | 3EKIA |
| 3ELBA | 3ELFA | 3ELSA | 3EMFA | 3EMIA |
| 3EMRA | 3EMVA | 3EN0A | 3ENUA | 3EO4A |
| 3EO6A | 3EO7A | 3EOIA | 3EOJA | 3EOQA |
| 3EQIA | 3EQXA | 3ER6A | 3ER7A | 3ES1A |
| 3ES4A | 3ESLA | 3ESSA | 3ETJA | 3ETTA |
| 3EU3A | 3EU7A | 3EWBX | 3EXZA | 3EYEA |
| 3EYTA | 3EZ2A | 3EZIA | 3EZUA | 3F0DA |
| 3F0PA | 3F1LA | 3F1PB | 3F1ZA | 3F2EA |
| 3F2ZA | 3F3KA | 3F40A | 3F43A | 3F44A |
| 3F4CA | 3F4MA | 3F5RA | 3F62A | 3F66A |
| 3F67A | 3F6VA | 3F6YA | 3F75P | 3F7CA |
| 3F7EA | 3F7QA | 3F7WA | 3F7XA | 3F83A |
| 3F8XA | 3F95A | 3F9TA | 3F9UA | 3F9XA |
| 3FB9A | 3FBLA | 3FBUA | 3FBZA | 3FCNA |
| 3FD3A | 3FD5A | 3FDHA | 3FDJA | 3FDSC |
| 3FDSD | 3FDXA | 3FDYA | 3FEDA | 3FEGA |
| 3FETA | 3FEYA | 3FF1A | 3FF5A | 3FFRA |
| 3FFVA | 3FG9A | 3FGHA | 3FGRA | 3FGRB |
| 3FGVA | 3FHDA | 3FHKA | 3FI1A | 3FI7A |
| 3FIAA | 3FIDA | 3FILA | 3FJ1A | 3FJSA |
| 3FJUB | 3FJVA | 3FKAA | 3FKCA | 3FKRA |
| 3FLBA | 3FLOA | 3FLOB | 3FM2A | 3FMCA |
| 3FMYA | 3FN2A | 3FN5A | 3FNBA | 3FO3A |
| 3FOTA | 3FOVA | 3FPFA | 3FPWA | 3FRHA |
| 3FRQA | 3FRRA | 3FSAA | 3FSEA | 3FSGA |
| 3FSOA | 3FSSA | 3FSUA | 3FT1A | 3FT7A |
| 3FUCA | 3FUWA | 3FVSA | 3FVVA | 3FVWA |
| 3FVYA | 3FW9A | 3FWBA | 3FWBB | 3FWKA |
| 3FWYA | 3FWZA | 3FX7A | 3FXAA | 3FXQA |
| 3FY3A | 3FY6A | 3FYBA | 3FYMA | 3FZ0A |
| 3G02A | 3G0KA | 3G0MA | 3G0TA | 3G1JA |
| 3G1WA | 3G21A | 3G23A | 3G27A | 3G2BA |
| 3G2EA | 3G2SA | 3G36A | 3G3OA | 3G3TA |
| 3G46A | 3G48A | 3G4EA | 3G5BA | 3G5SA |
| 3G5TA | 3G7GA | 3G7NA | 3G7QA | 3G7UA |
| 3G85A | 3G89A | 3G8YA | 3G91A | 3G9TA |
| 3G9XA | 3GA4A | 3GA8A | 3GAEA | 3GB5A |
| 3GBWA | 3GBYA | 3GD0A | 3GD4A | 3GDWA |
| 3GDZA | 3GE2A | 3GE3A | 3GE3B | 3GE3C |
| 3GE3E | 3GETA | 3GF6A | 3GF8A | 3GFAA |
| 3GFFA | 3GFPA | 3GG7A | 3GH1A | 3GI1A |
| 3GI7A | 3GIQA | 3GIUA | 3GIWA | 3GJ0A |
| 3GJYA | 3GK7A | 3GKEA | 3GKJA | 3GKNA |
| 3GKRA | 3GLVA | 3GMGA | 3GMIA | 3GMOA |
| 3GMXA | 3GN6A | 3GNEA | 3GNJA | 3GNLA |
| 3GNZP | 3GO5A | 3GO9A | 3GOCA | 3GOEA |
| 3GOHA | 3GONA | 3GP4A | 3GP6A | 3GPIA |
| 3GQQA | 3GR3A | 3GRLA | 3GS9A | 3GU3A |
| 3GUDA | 3GUUA | 3GV1A | 3GVEA | 3GVOA |
| 3GWIA | 3GWJA | 3GWNA | 3GWQA | 3GWRA |
| 3GX8A | 3GXHA | 3GXWA | 3GYCA | 3GYKA |
| 3GYZA | 3GZAA | 3GZBA | 3GZIA | 3GZKA |
| 3GZRA | 3GZTB | 3GZUA | 3H05A | 3H09A |
| 3H0NA | 3H0OA | 3H0UA | 3H0WA | 3H0WB |
| 3H14A | 3H16A | 3H1DA | 3H20A | 3H2HA |
| 3H2SA | 3H2ZA | 3H35A | 3H3IA | 3H3LA |
| 3H3WA | 3H4NA | 3H4TA | 3H4XA | 3H51A |
| 3H5JA | 3H5LA | 3H63A | 3H6JA | 3H6PA |
| 3H6PC | 3H6QA | 3H6RA | 3H74A | 3H79A |
| 3H7CX | 3H7HB | 3H7IA | 3H7JA | 3H7LA |
| 3H87A | 3H87C | 3H8DA | 3H8DE | 3H8GA |
| 3H8TA | 3H8VA | 3H9CA | 3H9MA | 3H9PA |
| 3H9WA | 3HA2A | 3HA9A | 3HBNA | 3HBZA |
| 3HC1A | 3HCZA | 3HDOA | 3HDXA | 3HE5A |
| 3HE5B | 3HE8A | 3HFA1 | 3HFTA | 3HFWA |
| 3HGTA | 3HGUA | 3HH1A | 3HHSA | 3HI0A |
| 3HIDA | 3HJ4A | 3HKMA | 3HKPA | 3HKWA |
| 3HL0A | 3HL1A | 3HLKA | 3HLXA | 3HLZA |
| 3HM4A | 3HN0A | 3HN5A | 3HNYM | 3HO6A |
| 3HOIA | 3HOLA | 3HP4A | 3HP7A | 3HPCX |
| 3HPDA | 3HQ1A | 3HQXA | 3HR0A | 3HRGA |
| 3HRLA | 3HRRA | 3HRZA | 3HRZB | 3HSAA |
| 3HSHA | 3HSIA | 3HSYA | 3HT1A | 3HTRA |
| 3HTYA | 3HUTA | 3HV2A | 3HV8A | 3HVVA |
| 3HVYA | 3HWPA | 3HWUA | 3HX3A | 3HX8A |
| 3HXJA | 3HXLA | 3HY0A | 3HYNA | 3HZ6A |
| 3HZ8A | 3HZPA | 3HZSA | 3I09A | 3I0ZA |
| 3I10A | 3I1AA | 3I23A | 3I2KA | 3I2VA |
| 3I45A | 3I48A | 3I4GA | 3I4OA | 3I4PA |
| 3I4ZA | 3I5TA | 3I6IA | 3I7AA | 3I7JA |
| 3I7MA | 3I84A | 3I94A | 3I9SA | 3IACA |
| 3IALA | 3IARA | 3IB5A | 3IC1A | 3IC3A |
| 3IC5A | 3ICVA | 3ID1A | 3IDFA | 3IDUA |
| 3IE4A | 3IEEA | 3IFNP | 3IG2A | 3IG3A |
| 3IG5A | 3IG9A | 3IGFA | 3IGHX | 3IGSA |
| 3IGZB | 3IHJA | 3IHVA | 3II2A | 3IISM |
| 3IIXA | 3IJLA | 3IJMA | 3IJWA | 3IK5A |
| 3IKBA | 3IKWA | 3ILSA | 3ILWA | 3ILXA |
| 3IM1A | 3IM3A | 3IM4C | 3IMKA | 3IMOA |
| 3INGA | 3IOHA | 3IP0A | 3IP4B | 3IP4C |
| 3IPFA | 3IPJA | 3IQ2A | 3IQUA | 3IR4A |
| 3IRAA | 3IRBA | 3IRPX | 3IRSA | 3ISAA |
| 3ISMC | 3ISQA | 3ISRA | 3ISXA | 3IT3A |
| 3IT5A | 3ITFA | 3ITQA | 3IU5A | 3IU6A |
| 3IUFA | 3IUKA | 3IUOA | 3IUWA | 3IUZA |
| 3IV0A | 3IV3A | 3IV4A | 3IVFA | 3IVVA |
| 3IWFA | 3IWGA | 3IX3A | 3IXLA | 3IYJA |
| 3IYLU | 3IYLW | 3IYMA | 3IYNN | 3IYNO |
| 3IYNQ | 3IZ0C | 3IZ0D | 3IZQ0 | 3IZQ1 |
| 3IZXD | 3J09A | 3J0GM | 3J0HA | 3J0JA |
| 3J0JH | 3J0JI | 3J0JJ | 3J0KA | 3J0KB |
| 3J0KC | 3J0KD | 3J0KE | 3J0KI | 3J0KJ |
| 3J0KK | 3J15A | 3J1NL | 3J1OI | 3J1OJ |
| 3J1OL | 3J1OM | 3J1TA | 3J1WA | 3J1ZP |
| 3J26A | 3J26N | 3J2MA | 3J2MU | 3J2VA |
| 3J2WE | 3J2WQ | 3J2YA | 3J31A | 3J3R1 |
| 3J40A | 3J40H | 3J47U | 3J47V | 3J4PA |
| 3J4UH | 3J5PA | 3JQ0A | 3JQ1A | 3JQHA |
| 3JRNA | 3JRVA | 3JS6A | 3JSBA | 3JTMA |
| 3JTWA | 3JTXA | 3JU0A | 3JU3A | 3JU4A |
| 3JU7A | 3JUDA | 3JUMA | 3JUWA | 3JVLA |
| 3JVOA | 3JX8A | 3JX9A | 3JXOA | 3JXPA |
| 3JYGA | 3JYHA | 3JYOA | 3JYZA | 3JZ0A |
| 3JZ9A | 3K05A | 3K0BA | 3K0LA | 3K0XA |
| 3K0ZA | 3K11A | 3K1QF | 3K1TA | 3K1UA |
| 3K1WA | 3K1ZA | 3K21A | 3K26A | 3K2OA |
| 3K2SA | 3K3CA | 3K3FA | 3K40A | 3K43A |
| 3K4IA | 3K50A | 3K5JA | 3K67A | 3K69A |
| 3K6IA | 3K6MA | 3K6OA | 3K6QA | 3K6YA |
| 3K7DA | 3K7IB | 3K8WA | 3K8XA | 3K93A |
| 3K94A | 3K9WA | 3KA2A | 3KB2A | 3KB9A |
| 3KBGA | 3KBLA | 3KBRA | 3KBYA | 3KC2A |
| 3KD3A | 3KD4A | 3KDFA | 3KDFB | 3KDGA |
| 3KDWA | 3KE3A | 3KE7A | 3KEOA | 3KESA |
| 3KEVA | 3KFFA | 3KFOA | 3KG0A | 3KG8A |
| 3KG9A | 3KGDA | 3KGKA | 3KGWA | 3KH1A |
| 3KHIA | 3KIZA | 3KK4A | 3KKEA | 3KKFA |
| 3KKGA | 3KL7A | 3KLDA | 3KLQA | 3KM5A |
| 3KMIA | 3KMVA | 3KNDB | 3KOGA | 3KORA |
| 3KOTA | 3KP1A | 3KP1E | 3KPEA | 3KQ5A |
| 3KRAB | 3KRTA | 3KS3A | 3KS6A | 3KSMA |
| 3KSNA | 3KSXA | 3KT7A | 3KTAA | 3KTAB |
| 3KU4A | 3KUCB | 3KUUA | 3KUVA | 3KVCA |
| 3KVHA | 3KVPA | 3KWEA | 3KWLA | 3KWOA |
| 3KWRA | 3KWSA | 3KXDA | 3KXRA | 3KXWA |
| 3KYFA | 3KYJA | 3KYZA | 3KZ3A | 3KZ5A |
| 3KZPA | 3KZSA | 3L00A | 3L07A | 3L0FA |
| 3L0VA | 3L12A | 3L15A | 3L1WA | 3L22A |
| 3L32A | 3L39A | 3L46A | 3L4AA | 3L4HA |
| 3L51B | 3L5IA | 3L5LA | 3L60A | 3L6BA |
| 3L6GA | 3L6TA | 3L7HA | 3L8QA | 3L8WA |
| 3L91B | 3L9AX | 3LAAA | 3LAEA | 3LAGA |
| 3LATA | 3LAXA | 3LB2A | 3LB5A | 3LBMA |
| 3LC3A | 3LCCA | 3LD01 | 3LD7A | 3LDCA |
| 3LDFA | 3LE4A | 3LEDA | 3LETA | 3LEWA |
| 3LFJA | 3LFPA | 3LFRA | 3LFTA | 3LGBA |
| 3LHCA | 3LHEA | 3LHIA | 3LHNA | 3LHOA |
| 3LHRA | 3LHSA | 3LICA | 3LIDA | 3LIUA |
| 3LJBA | 3LJUX | 3LJXA | 3LKBA | 3LKDA |
| 3LKMA | 3LKVA | 3LL3A | 3LL5A | 3LL7A |
| 3LLBA | 3LLHA | 3LLOA | 3LLPA | 3LLUA |
| 3LLXA | 3LM2A | 3LM3A | 3LM6A | 3LMZA |
| 3LNLA | 3LNOA | 3LO8A | 3LOFA | 3LOGA |
| 3LOPA | 3LPWA | 3LPZA | 3LQ9A | 3LQBA |
| 3LQCA | 3LQKA | 3LRQA | 3LS9A | 3LSNA |
| 3LSSA | 3LT0A | 3LTJA | 3LU0C | 3LU0D |
| 3LU0E | 3LUFA | 3LULA | 3LURA | 3LUUA |
| 3LUYA | 3LVUA | 3LWCA | 3LWGA | 3LWSA |
| 3LWXA | 3LX3A | 3LXRF | 3LY1A | 3LYDA |
| 3LYEA | 3LYGA | 3LYHA | 3LYYA | 3LZAA |
| 3LZKA | 3LZQA | 3LZWA | 3M0MA | 3M0ZA |
| 3M1IB | 3M1RA | 3M1TA | 3M1UA | 3M1XA |
| 3M2TA | 3M33A | 3M3PA | 3M4RA | 3M5QA |
| 3M66A | 3M6JA | 3M6WA | 3M6ZA | 3M73A |
| 3M7AA | 3M7OA | 3M7PA | 3M84A | 3M8JA |
| 3M9QA | 3M9VA | 3MA0A | 3MA2B | 3MABA |
| 3MALA | 3MAOA | 3MAYA | 3MBBA | 3MBRX |
| 3MC3A | 3MC9A | 3MCBA | 3MCBB | 3MCQA |
| 3MCWA | 3MCXA | 3MCZA | 3MD7A | 3MD9A |
| 3MDMA | 3MDPA | 3MDQA | 3MDUA | 3ME0B |
| 3ME7A | 3MEAA | 3MEMA | 3MF7A | 3MG1A |
| 3MG9A | 3MHSB | 3MHSC | 3MHXA | 3MI2A |
| 3MILA | 3MJFA | 3MJOA | 3MK4A | 3MK6A |
| 3MKHA | 3MKOA | 3ML1A | 3ML3A | 3MMGA |
| 3MMHA | 3MMLB | 3MOIA | 3MOZA | 3MP2A |
| 3MPCA | 3MPKA | 3MQDA | 3MQZA | 3MR0A |
| 3MSHA | 3MSWA | 3MSXB | 3MT0A | 3MT1A |
| 3MU7A | 3MUJA | 3MUQA | 3MVCA | 3MVKA |
| 3MVRA | 3MVSA | 3MVUA | 3MW6A | 3MW8A |
| 3MWDA | 3MWPA | 3MWXA | 3MWZA | 3MX1A |
| 3MX7A | 3MXNA | 3MXNB | 3MXOA | 3MXZA |
| 3MY2A | 3MYXA | 3MZ0A | 3MZ2A | 3MZFA |
| 3MZIA | 3MZOA | 3N01A | 3N08A | 3N0AA |
| 3N0KA | 3N0RA | 3N0UA | 3N0XA | 3N17A |
| 3N1EA | 3N1MC | 3N2OA | 3N2WA | 3N37A |
| 3N3RA | 3N4JA | 3N5KA | 3N6XA | 3N6YA |
| 3N6ZA | 3N72A | 3N75A | 3N77A | 3N79A |
| 3N8BA | 3N91A | 3N9BA | 3N9KA | 3NA1A |
| 3NA6A | 3NAPA | 3NAPB | 3NAPC | 3NB2A |
| 3NBCA | 3NBMA | 3NCEA | 3NCEB | 3NCTA |
| 3NDQA | 3NE0A | 3NE8A | 3NECA | 3NFIA |
| 3NFTA | 3NFWA | 3NGGA | 3NGWA | 3NHEA |
| 3NHIA | 3NIRA | 3NJCA | 3NJEA | 3NJNA |
| 3NK7A | 3NKEA | 3NKGA | 3NKLA | 3NKUA |
| 3NKZA | 3NMDA | 3NMWA | 3NN1A | 3NNBA |
| 3NO0A | 3NO2A | 3NO7A | 3NOHA | 3NOIA |
| 3NOJA | 3NOQA | 3NPDA | 3NPFA | 3NPKA |
| 3NPPA | 3NQHA | 3NQIA | 3NQNA | 3NQZA |
| 3NR5A | 3NREA | 3NRFA | 3NRHA | 3NRSA |
| 3NRWA | 3NS6A | 3NSWA | 3NSXA | 3NT8A |
| 3NUAA | 3NUFA | 3NUQA | 3NV0A | 3NV0B |
| 3NVSA | 3NVWB | 3NVXA | 3NW4A | 3NWAA |
| 3NWOA | 3NY3A | 3NYHA | 3NYMA | 3NYTA |
| 3NZEA | 3NZKA | 3NZLA | 3NZNA | 3O0DA |
| 3O0FA | 3O0QA | 3O0YA | 3O10A | 3O14A |
| 3O1NA | 3O2EA | 3O2GA | 3O2TA | 3O3MA |
| 3O3MB | 3O3YA | 3O48A | 3O4NA | 3O4PA |
| 3O53A | 3O6CA | 3O6NA | 3O6PA | 3O6QA |
| 3O6QB | 3O7BA | 3O83A | 3O8MA | 3O8QA |
| 3O8ZA | 3O94A | 3O9ZA | 3OA1A | 3OA8A |
| 3OA8B | 3OAJA | 3OAMA | 3OBEA | 3OBFA |
| 3OBHA | 3OBLA | 3OC8A | 3OCJA | 3OCMA |
| 3OCOA | 3OCRA | 3OCUA | 3OD1A | 3OD9A |
| 3ODTA | 3OEPA | 3OF1A | 3OF4A | 3OFGA |
| 3OFKA | 3OG2A | 3OG6A | 3OG6B | 3OG9A |
| 3OGHA | 3OGNA | 3OH3A | 3OH8A | 3OHEA |
| 3OHGA | 3OIGA | 3OIOA | 3OIRA | 3OISA |
| 3OIZA | 3OJ0A | 3OJCA | 3OKAA | 3OKGA |
| 3OKQA | 3OKXA | 3OKYA | 3OKYB | 3OL3A |
| 3OLOA | 3OLQA | 3OM0A | 3OMDA | 3OMYA |
| 3ON7A | 3ON9A | 3ONDA | 3ONHA | 3ONJA |
| 3ONOA | 3ONPA | 3ONQA | 3OO8A | 3OOSA |
| 3OOUA | 3OOXA | 3OP6A | 3OP8A | 3OQIA |
| 3OQPA | 3OQQA | 3OR1A | 3OR1B | 3ORJA |
| 3ORUA | 3OS4A | 3OS6A | 3OSEA | 3OSJA |
| 3OSTA | 3OT9A | 3OTIA | 3OTNA | 3OTTA |
| 3OUIA | 3OULA | 3OUVA | 3OUXB | 3OV5A |
| 3OV8A | 3OVKA | 3OVWA | 3OWRA | 3OXHA |
| 3OXPA | 3OYVA | 3P02A | 3P06A | 3P0BA |
| 3P0FA | 3P0YA | 3P1VA | 3P24A | 3P2CA |
| 3P2EA | 3P2TA | 3P3AA | 3P3CA | 3P42A |
| 3P4GA | 3P4HA | 3P51A | 3P6DA | 3P6LA |
| 3P8CA | 3P8CB | 3P8CD | 3P8CF | 3P97A |
| 3P9AA | 3P9VA | 3PASA | 3PB6X | 3PBTA |
| 3PC7A | 3PCVA | 3PD7A | 3PE6A | 3PE7A |
| 3PE9A | 3PESA | 3PETA | 3PF0A | 3PF6A |
| 3PF7A | 3PFEA | 3PFGA | 3PFOA | 3PFTA |
| 3PFYA | 3PG0A | 3PG6A | 3PGUA | 3PHXA |
| 3PHXB | 3PHZA | 3PI7A | 3PICA | 3PIJA |
| 3PIKA | 3PIUA | 3PIVA | 3PIWA | 3PJPA |
| 3PJXA | 3PKOA | 3PL0A | 3PL2A | 3PLWA |
| 3PM2A | 3PM6A | 3PMCA | 3PMDA | 3PMEA |
| 3PMMA | 3PMOA | 3PMSA | 3PN3A | 3PNLB |
| 3PNNA | 3PNXA | 3POHA | 3POJA | 3POPA |
| 3POWA | 3PP2A | 3PP5A | 3PPGA | 3PPLA |
| 3PPRA | 3PQHA | 3PR6A | 3PRNA | 3PRYA |
| 3PS0A | 3PSEB | 3PSMA | 3PT1A | 3PT8A |
| 3PT9A | 3PU5A | 3PU9A | 3PUAA | 3PUCA |
| 3PVCA | 3PVEA | 3PVHA | 3PVJA | 3PVKA |
| 3PVTA | 3PVZA | 3PW3A | 3PWKA | 3PXLA |
| 3PXPA | 3PYWA | 3PZFA | 3PZSA | 3PZWA |
| 3Q0IA | 3Q0YA | 3Q12A | 3Q18A | 3Q1CA |
| 3Q1IA | 3Q1NA | 3Q20A | 3Q2UA | 3Q39A |
| 3Q3EA | 3Q46A | 3Q49B | 3Q4OA | 3Q62A |
| 3Q64A | 3Q6SA | 3Q6VA | 3Q7CA | 3Q7MA |
| 3Q7RA | 3Q8DA | 3Q8GA | 3Q9NA | 3Q9VA |
| 3QAOA | 3QAVA | 3QB4B | 3QB8A | 3QC0A |
| 3QC5X | 3QC7A | 3QCPA | 3QDLA | 3QDPA |
| 3QEKA | 3QF7C | 3QFMA | 3QGUA | 3QH6A |
| 3QHBA | 3QHEB | 3QHOA | 3QHPA | 3QHYB |
| 3QI7A | 3QIOA | 3QISA | 3QL9A | 3QLEA |
| 3QM9A | 3QNMA | 3QNSA | 3QOOA | 3QORA |
| 3QOWA | 3QPAA | 3QQ8B | 3QR7A | 3QRAA |
| 3QRLA | 3QS2A | 3QSJA | 3QSQA | 3QTAA |
| 3QTHA | 3QTMA | 3QU3A | 3QU5A | 3QUFA |
| 3QUVA | 3QVAA | 3QVEA | 3QVPA | 3QVQA |
| 3QVTA | 3QW9A | 3QWGA | 3QWNA | 3QWOC |
| 3QWPA | 3QXFA | 3QXLA | 3QXYA | 3QXZA |
| 3QY3A | 3QYEA | 3QYFA | 3QYJA | 3QZ0A |
| 3QZBA | 3QZMA | 3QZRA | 3QZXA | 3R0VA |
| 3R1KA | 3R1XA | 3R26A | 3R2QA | 3R41A |
| 3R4IA | 3R4KA | 3R4VA | 3R4ZA | 3R5TA |
| 3R5ZA | 3R6DA | 3R6UA | 3R72A | 3R75A |
| 3R7GA | 3R84A | 3R84B | 3R87A | 3R89A |
| 3R8JA | 3R9FA | 3R9MA | 3RAOA | 3RAUA |
| 3RBSA | 3RCOA | 3RCQA | 3RD7A | 3RDOA |
| 3REAA | 3RF3A | 3RF7A | 3RGQA | 3RHFA |
| 3RHTA | 3RHZA | 3RJOA | 3RJTA | 3RJUA |
| 3RJVA | 3RKCA | 3RKGA | 3RKLA | 3RKXA |
| 3RL5A | 3RLEA | 3RLGA | 3RLKA | 3RLOA |
| 3RLSA | 3RM3A | 3RMQA | 3RNLA | 3RNQA |
| 3RNRA | 3RO3A | 3ROBA | 3ROFA | 3RPCA |
| 3RPDA | 3RPJA | 3RPPA | 3RQ1A | 3RQ9A |
| 3RQTA | 3RRCA | 3RRLA | 3RRLB | 3RRXA |
| 3RT2A | 3RT3C | 3RT9A | 3RTLA | 3RU6A |
| 3RUIA | 3RUVA | 3RV0A | 3RV1A | 3RVAA |
| 3RVCA | 3RWNA | 3RX6A | 3RX9A | 3RZIA |
| 3RZNA | 3S06A | 3S0AA | 3S18A | 3S1XA |
| 3S21A | 3S25A | 3S2JA | 3S2RA | 3S3TA |
| 3S44A | 3S4EA | 3S5BA | 3S5MA | 3S5QA |
| 3S5TA | 3S5WA | 3S6EA | 3S6FA | 3S6SA |
| 3S6WA | 3S7OA | 3S83A | 3S8GA | 3S8GB |
| 3S8IA | 3S8MA | 3S90C | 3S9JA | 3S9XA |
| 3S9ZA | 3SB1A | 3SB4A | 3SBMA | 3SBTB |
| 3SBZA | 3SC7X | 3SCYA | 3SD2A | 3SD6A |
| 3SDEA | 3SDOA | 3SEEA | 3SFVB | 3SG0A |
| 3SGGA | 3SGHA | 3SGWA | 3SHGA | 3SHGB |
| 3SHOA | 3SHPA | 3SHQA | 3SIGA | 3SJ5A |
| 3SJHB | 3SK2A | 3SK7A | 3SK9A | 3SKQA |
| 3SKVA | 3SL9C | 3SLRA | 3SNKA | 3SO6A |
| 3SOJA | 3SONA | 3SOVA | 3SOYA | 3SP7A |
| 3SPAA | 3SQZA | 3SR0A | 3SREA | 3SS7X |
| 3SSBC | 3SU6A | 3SUKA | 3SUMA | 3SUUA |
| 3SW0X | 3SWOA | 3SWYA | 3SX6A | 3SXUA |
| 3SXUB | 3SXYA | 3SY1A | 3SY6A | 3SZ3A |
| 3SZVA | 3SZYA | 3T0HA | 3T0PA | 3T12A |
| 3T12B | 3T2CA | 3T30A | 3T33A | 3T36A |
| 3T3LA | 3T3PA | 3T3PB | 3T47A | 3T4LA |
| 3T63M | 3T6AA | 3T6GB | 3T6OA | 3T6PA |
| 3T6QA | 3T6SA | 3T74A | 3T7AA | 3T7DA |
| 3T7HA | 3T7LA | 3T8BA | 3T8JA | 3T8KA |
| 3T8XA | 3T90A | 3T92A | 3T94A | 3T9GA |
| 3T9WA | 3T9YA | 3TACB | 3TAWA | 3TB6A |
| 3TBDA | 3TBKA | 3TC3A | 3TC5A | 3TC8A |
| 3TCQA | 3TCVA | 3TDQA | 3TDSA | 3TDWA |
| 3TDZC | 3TE4A | 3TE8A | 3TEUA | 3TEWA |
| 3TFGA | 3TFJA | 3TG0A | 3TG2A | 3TG7A |
| 3TG9A | 3TGHA | 3TH2T | 3THMF | 3TIJA |
| 3TIPA | 3TIWA | 3TJ8A | 3TJMA | 3TJYA |
| 3TK0A | 3TKJA | 3TKMA | 3TKTA | 3TL1A |
| 3TL8B | 3TLQA | 3TM8A | 3TN2A | 3TNLA |
| 3TNYA | 3TO3A | 3TO7A | 3TOJA | 3TOSA |
| 3TOWA | 3TR2A | 3TS3A | 3TS9A | 3TSRE |
| 3TSSA | 3TT9A | 3TTCA | 3TTGA | 3TTYA |
| 3TU8A | 3TUFA | 3TUFB | 3TUOA | 3TVAA |
| 3TVJA | 3TVKA | 3TVZA | 3TXSA | 3TY1A |
| 3TYSA | 3TYTA | 3TZTA | 3U02A | 3U07A |
| 3U0HA | 3U0IA | 3U1DA | 3U1KA | 3U1WA |
| 3U24A | 3U2GA | 3U2UA | 3U3EA | 3U3GA |
| 3U3LC | 3U3ZA | 3U49A | 3U4GA | 3U4VA |
| 3U52C | 3U52E | 3U64A | 3U65A | 3U6GA |
| 3U7QA | 3U7QB | 3U7RA | 3U7ZA | 3U81A |
| 3U83A | 3U8UA | 3U8VA | 3U95A | 3U97A |
| 3U99A | 3U9GA | 3U9JA | 3U9RB | 3U9WA |
| 3UAFA | 3UANA | 3UAWA | 3UB1A | 3UB6A |
| 3UCIA | 3UCPA | 3UCSA | 3UD1A | 3UDFA |
| 3UEJA | 3UENA | 3UF6A | 3UF7A | 3UFBA |
| 3UFEA | 3UFIA | 3UGUA | 3UI4A | 3UIDA |
| 3UIPD | 3UITA | 3UJCA | 3UJKA | 3ULJA |
| 3ULLA | 3ULTA | 3UMOA | 3UOAB | 3UP3A |
| 3UPLA | 3UPSA | 3UQ8A | 3UQSA | 3UQYS |
| 3UR8A | 3URRA | 3US3A | 3USHA | 3USTA |
| 3UUEA | 3UULA | 3UUWA | 3UV0A | 3UW1A |
| 3UW3A | 3UWSA | 3UWSB | 3UX2A | 3UXFA |
| 3UXJA | 3UYXA | 3UZQB | 3V0DA | 3V0RA |
| 3V1AA | 3V1VA | 3V2UA | 3V33A | 3V39A |
| 3V3LA | 3V46A | 3V48A | 3V4KA | 3V4YB |
| 3V5CA | 3V5UA | 3V5WB | 3V5WG | 3V68A |
| 3V6OA | 3V75A | 3V7BA | 3V7DA | 3V7NA |
| 3V85A | 3VA4A | 3VA9A | 3VATA | 3VAVA |
| 3VBCA | 3VC1A | 3VCXA | 3VDHA | 3VDJA |
| 3VEJA | 3VENA | 3VF7A | 3VFBA | 3VG7A |
| 3VGIA | 3VGLA | 3VH5W | 3VHLA | 3VHVA |
| 3VIIA | 3VISA | 3VJ9A | 3VJZA | 3VK0A |
| 3VK5A | 3VKJA | 3VKWA | 3VL1A | 3VLAA |
| 3VMKA | 3VMNA | 3VN0A | 3VN3A | 3VNBA |
| 3VNIA | 3VNRA | 3VNYA | 3VOQA | 3VORA |
| 3VOTA | 3VPBA | 3VPBE | 3VQFA | 3VQJA |
| 3VR0A | 3VRDB | 3VRPA | 3VSJA | 3VSJB |
| 3VSNA | 3VSSA | 3VSVA | 3VU0A | 3VU9A |
| 3VU9B | 3VUBA | 3VUPA | 3VV1A | 3VVVA |
| 3VVYA | 3VWAA | 3VWIA | 3VWNX | 3VX0A |
| 3VXCA | 3VXJA | 3VYGA | 3VYGB | 3VYWA |
| 3VZ9B | 3VZ9D | 3VZHA | 3VZXA | 3W06A |
| 3W07A | 3W0KA | 3W0OA | 3W0TA | 3W15B |
| 3W19C | 3W1OA | 3W4SA | 3W52A | 3W54A |
| 3W57A | 3W5HA | 3W5NA | 3W6DA | 3W6SA |
| 3W6WA | 3W7TA | 3W8JA | 3W9DA | 3W9KA |
| 3W9SA | 3WA2X | 3WA5B | 3WAJA | 3WARA |
| 3WASA | 3WBNA | 3WBZA | 3WC4A | 3WCQA |
| 3WDCA | 3WDNA | 3WEUA | 3WG3A | 3WH2A |
| 3WITA | 3WJPA | 3WKGA | 3ZBDA | 3ZBGA |
| 3ZBIA | 3ZBIB | 3ZBIC | 3ZBJA | 3ZBOA |
| 3ZD0A | 3ZD8A | 3ZDMC | 3ZE3A | 3ZEDD |
| 3ZEEA | 3ZEUA | 3ZEUB | 3ZF8A | 3ZFIA |
| 3ZFJA | 3ZFPA | 3ZFZA | 3ZG4A | 3ZG7A |
| 3ZGIA | 3ZGOA | 3ZH6A | 3ZH9B | 3ZHIA |
| 3ZIDA | 3ZIEA | 3ZIHA | 3ZIKA | 3ZIUA |
| 3ZJ0A | 3ZJ1A | 3ZJ2A | 3ZJBA | 3ZJEA |
| 3ZK9A | 3ZL8A | 3ZLCA | 3ZN4A | 3ZN6A |
| 3ZNUA | 3ZNVA | 3ZOJA | 3ZOQB | 3ZPJA |
| 3ZPMA | 3ZPNA | 3ZQOA | 3ZQSA | 3ZQUA |
| 3ZQXA | 3ZR0A | 3ZR8X | 3ZRGA | 3ZRIA |
| 3ZRXA | 3ZSJA | 3ZSUA | 3ZT9A | 3ZTPA |
| 3ZUAA | 3ZUCA | 3ZUIA | 3ZUZA | 3ZVLA |
| 3ZVVA | 3ZVWA | 3ZW6A | 3ZWFA | 3ZWHQ |
| 3ZWLB | 3ZWLE | 3ZX2A | 3ZX4A | 3ZX8A |
| 3ZXCA | 3ZXKA | 3ZXNA | 3ZY7A | 3ZYLA |
| 3ZYPA | 3ZYQA | 3ZYTA | 3ZZHA | 3ZZOA |
| 3ZZPA | 3ZZSA | 487DI | 487DJ | 487DN |
| 4A01A | 4A02A | 4A0GA | 4A27A | 4A29A |
| 4A2BA | 4A2VA | 4A35A | 4A37A | 4A3PA |
| 4A4AA | 4A4JA | 4A54B | 4A56A | 4A57A |
| 4A5LA | 4A5QA | 4A5SA | 4A5UA | 4A5UB |
| 4A5VA | 4A5ZA | 4A69C | 4A6DA | 4A6JA |
| 4A6QA | 4A7FB | 4A7UA | 4A7WA | 4A8XB |
| 4A9VA | 4A9ZA | 4AA7A | 4AAIA | 4AAZA |
| 4ABLA | 4AC1X | 4AC7A | 4AC7B | 4AC7C |
| 4ACFA | 4ACJA | 4ACKA | 4ACOA | 4ACVA |
| 4ACYA | 4ADIA | 4ADNA | 4ADZA | 4AE2A |
| 4AE4A | 4AE7A | 4AF8A | 4AFFA | 4AFKA |
| 4AFMA | 4AGHA | 4AHQA | 4AIVA | 4AIWA |
| 4AJSA | 4AJYB | 4AJYC | 4AJYV | 4AK2A |
| 4AKLA | 4AKZA | 4AL0A | 4ALZA | 4AMMA |
| 4ANNA | 4AO6A | 4APOA | 4APWA | 4AQ1A |
| 4AQ4A | 4AQOA | 4AR0A | 4AR9A | 4ARUA |
| 4ASCA | 4ASMB | 4AT7B | 4ATEA | 4ATGA |
| 4ATHA | 4ATMA | 4ATTA | 4AU1A | 4AU6A |
| 4AUKA | 4AV2A | 4AV2M | 4AVAA | 4AVRA |
| 4AVSA | 4AW7A | 4AW9A | 4AWNA | 4AX1B |
| 4AXDA | 4AXOA | 4AXVA | 4AY0A | 4AY7A |
| 4AYOA | 4AZ3B | 4AZ6A | 4B0HA | 4B0MA |
| 4B0MB | 4B0TA | 4B0ZA | 4B1MA | 4B1YB |
| 4B29A | 4B2NA | 4B2OA | 4B2QT | 4B2QU |
| 4B2QV | 4B2QW | 4B2ZA | 4B3BA | 4B3FX |
| 4B4CA | 4B5OA | 4B5ZA | 4B62A | 4B6GA |
| 4B6XA | 4B7LA | 4B87A | 4B89A | 4B8AB |
| 4B8EA | 4B8XA | 4B8YA | 4B9GA | 4BA0A |
| 4BA1A | 4BA1B | 4BA1I | 4BA8A | 4BB9A |
| 4BBQA | 4BC3A | 4BCUA | 4BE3A | 4BEHB |
| 4BF3A | 4BFOA | 4BG2A | 4BGBA | 4BGCA |
| 4BGNA | 4BGOA | 4BGPA | 4BH5A | 4BHNA |
| 4BHQA | 4BHUA | 4BI8B | 4BIFA | 4BIJA |
| 4BINA | 4BIQA | 4BJIA | 4BJJA | 4BJJB |
| 4BJSA | 4BJTA | 4BK7A | 4BL0B | 4BLFA |
| 4BLPA | 4BLUA | 4BMDA | 4BN4A | 4BNDA |
| 4BOQA | 4BOUA | 4BPSA | 4BPZA | 4BQ2A |
| 4BQHA | 4BQNA | 4BRCA | 4BSVA | 4BSXA |
| 4BT1A | 4BT7A | 4BT9A | 4BTGA | 4BU0A |
| 4BUHA | 4BUUA | 4BV4L | 4BV4R | 4BVQA |
| 4BWCA | 4BWOA | 4BWRA | 4BWVA | 4BX4A |
| 4BX8A | 4BXHA | 4BZAA | 4BZIA | 4BZJA |
| 4BZKB | 4C12A | 4C24A | 4C2LA | 4C2OA |
| 4C3SA | 4C45A | 4C4PB | 4C58A | 4C6AA |
| 4C8DA | 4C91A | 4C97A | 4C9RA | 4C9RB |
| 4CA1A | 4CC9A | 4CC9B | 4CCDA | 4CDPA |
| 4D86A | 4D8BA | 4D9BA | 4D9IA | 4D9OA |
| 4D9SA | 4DAMA | 4DB5A | 4DCAA | 4DD5A |
| 4DDPA | 4DE9A | 4DEXA | 4DEXB | 4DF3A |
| 4DGUA | 4DHKA | 4DHLA | 4DI9A | 4DIFA |
| 4DIQA | 4DJAA | 4DJDD | 4DK2A | 4DKAC |
| 4DKCA | 4DKKA | 4DKNA | 4DKWA | 4DLHA |
| 4DLQA | 4DM5A | 4DMIA | 4DMVA | 4DMZA |
| 4DN7A | 4DNYA | 4DO3A | 4DO4A | 4DO7A |
| 4DOIA | 4DOOA | 4DOYA | 4DPZX | 4DQ6A |
| 4DQAA | 4DQJA | 4DRIB | 4DS7E | 4DT4A |
| 4DT5A | 4DTHA | 4DUHA | 4DUIA | 4DUNA |
| 4DUQA | 4DV8A | 4DVCA | 4DVEA | 4DW3A |
| 4DWDA | 4DWEA | 4DWRA | 4DX7A | 4DXAB |
| 4DXTA | 4DYQA | 4DZ1A | 4DZIA | 4DZOA |
| 4E05I | 4E15A | 4E1BA | 4E1PA | 4E1SA |
| 4E2UA | 4E2VA | 4E2ZA | 4E3EA | 4E3XA |
| 4E3YA | 4E44A | 4E44B | 4E4RA | 4E4WA |
| 4E4WB | 4E5VA | 4E6FA | 4E6KG | 4E6NB |
| 4E6WA | 4E70A | 4E72A | 4E9EA | 4E9LA |
| 4E9SA | 4EA9A | 4EACA | 4EADA | 4EAEA |
| 4EAIA | 4EAIB | 4EAIC | 4EBGA | 4EBYA |
| 4ECFA | 4EDGA | 4EDHA | 4EDPA | 4EE6A |
| 4EFHB | 4EFIA | 4EFOA | 4EFPA | 4EG0A |
| 4EG2A | 4EGCB | 4EGDA | 4EGOA | 4EGUA |
| 4EH1A | 4EHSA | 4EHUA | 4EHXA | 4EI0A |
| 4EICA | 4EIJA | 4EIRA | 4EIUA | 4EJOA |
| 4EJQA | 4EKFA | 4EKXA | 4EL6A | 4ELNA |
| 4ELYA | 4EMDA | 4EMNA | 4EMTA | 4ENFA |
| 4EO0A | 4EO1A | 4EO3A | 4EO7A | 4EOJB |
| 4EP4A | 4EPIA | 4EPSA | 4EPZA | 4EQ8A |
| 4EQAC | 4EQBA | 4EQLA | 4EQPA | 4EQQA |
| 4EQSA | 4ERCA | 4ERRA | 4ERYA | 4ES1A |
| 4ES8A | 4ESKA | 4ESMA | 4ESQA | 4ESUA |
| 4ESWA | 4ETNA | 4EU9A | 4EUOA | 4EV1A |
| 4EVUA | 4EVWA | 4EVZA | 4EW5A | 4EW7A |
| 4EXJA | 4EXKA | 4EXOA | 4EXRA | 4EYCA |
| 4EYSA | 4EYZA | 4EZ8A | 4EZGA | 4EZIA |
| 4F01A | 4F06A | 4F0JA | 4F0ZC | 4F1JA |
| 4F1VA | 4F1WA | 4F23A | 4F27A | 4F2EA |
| 4F2LA | 4F3LB | 4F3NA | 4F3VA | 4F4HA |
| 4F54A | 4F55A | 4F67A | 4F6TB | 4F7HA |
| 4F7UA | 4F7UP | 4F87A | 4F8BA | 4F8CA |
| 4F8EA | 4F8LA | 4F98A | 4F99A | 4F99B |
| 4F9DA | 4F9TA | 4F9ZA | 4FASD | 4FBCA |
| 4FBJA | 4FBRA | 4FBSA | 4FCHA | 4FCJA |
| 4FD9A | 4FDBA | 4FDWA | 4FDZA | 4FE3A |
| 4FFUA | 4FGLA | 4FGMA | 4FGQA | 4FH0A |
| 4FIXA | 4FK1A | 4FK9A | 4FKEA | 4FKLA |
| 4FKZA | 4FLBA | 4FMPA | 4FMRA | 4FMTA |
| 4FN7A | 4FNVA | 4FO5A | 4FP5D | 4FQGA |
| 4FQIB | 4FQNA | 4FR9A | 4FS7A | 4FS8A |
| 4FTDA | 4FTFA | 4FTXA | 4FUSA | 4FVGA |
| 4FVQA | 4FVYA | 4FW9A | 4FWWA | 4FX5A |
| 4FXIA | 4FYBA | 4FYKA | 4FYPA | 4FYYB |
| 4FZLA | 4FZPA | 4FZVB | 4G08A | 4G0IA |
| 4G0XA | 4G10A | 4G1IA | 4G1OA | 4G1QA |
| 4G22A | 4G26A | 4G29A | 4G2AA | 4G2UA |
| 4G2VA | 4G38A | 4G3NA | 4G3OA | 4G3VA |
| 4G4KA | 4G4SJ | 4G4SM | 4G4SN | 4G4SO |
| 4G4SP | 4G50A | 4G54A | 4G55A | 4G5AA |
| 4G6HA | 4G6UA | 4G78A | 4G79A | 4G7NA |
| 4G91A | 4G9EA | 4G9PA | 4G9SA | 4G9SB |
| 4GA2A | 4GAIA | 4GAKA | 4GAXA | 4GB5A |
| 4GBFA | 4GBMA | 4GC1A | 4GC3A | 4GC5A |
| 4GC8A | 4GCNA | 4GDIA | 4GDZA | 4GE6A |
| 4GEIA | 4GF0A | 4GF3A | 4GF3B | 4GFRA |
| 4GGJA | 4GGZA | 4GH9A | 4GHBA | 4GHGA |
| 4GHNA | 4GI3C | 4GI7A | 4GIEA | 4GIMA |
| 4GIOA | 4GJ4A | 4GJZA | 4GKHA | 4GL3A |
| 4GLKA | 4GLQA | 4GMFA | 4GMQA | 4GMUA |
| 4GN4B | 4GNEA | 4GNRA | 4GOFA | 4GOQA |
| 4GOUA | 4GPVA | 4GQ4A | 4GQBA | 4GQBB |
| 4GQRA | 4GQUA | 4GRNA | 4GS1A | 4GS3A |
| 4GS5A | 4GS7A | 4GS7B | 4GS7C | 4GT8A |
| 4GT9A | 4GUCA | 4GUTB | 4GV2A | 4GV5A |
| 4GVFA | 4GVQA | 4GWBA | 4GWGA | 4GWMA |
| 4GXBA | 4GXBB | 4GXTA | 4GXWA | 4GYIA |
| 4GYOA | 4GYTA | 4GYWA | 4GZKA | 4H08A |
| 4H0AA | 4H0CA | 4H14A | 4H1BA | 4H2HA |
| 4H2KA | 4H2WA | 4H2WC | 4H3TA | 4H3VA |
| 4H3WA | 4H41A | 4H4DA | 4H4JA | 4H4NA |
| 4H4VA | 4H59A | 4H5BA | 4H5IA | 4H5SB |
| 4H6CA | 4H6QA | 4H75A | 4H7LA | 4H7NA |
| 4H7WA | 4H7YA | 4H8EA | 4H9JA | 4H9RC |
| 4HAMA | 4HATB | 4HATC | 4HB9A | 4HBQA |
| 4HC5A | 4HCJA | 4HCSA | 4HD1A | 4HD5A |
| 4HDDA | 4HDRB | 4HE6A | 4HEGA | 4HEOA |
| 4HESA | 4HF0A | 4HFQA | 4HFSA | 4HFVA |
| 4HG2A | 4HGUA | 4HH3A | 4HH3C | 4HH5A |
| 4HHJA | 4HHRA | 4HHUA | 4HHVA | 4HI0B |
| 4HI0E | 4HI8B | 4HIAA | 4HITA | 4HJHA |
| 4HJJH | 4HKGA | 4HKHA | 4HL2A | 4HLBA |
| 4HLJA | 4HLSA | 4HMSA | 4HNLA | 4HNOA |
| 4HQ1A | 4HQZA | 4HR3A | 4HR6A | 4HROA |
| 4HRZA | 4HS1A | 4HS2A | 4HSPA | 4HSQA |
| 4HSTB | 4HTGA | 4HTLA | 4HTPA | 4HVKA |
| 4HW6A | 4HWMA | 4HWNA | 4HWSA | 4HWVA |
| 4HY4A | 4HYEA | 4HYQA | 4HYRA | 4HYZA |
| 4HZ4A | 4HZ9A | 4HZ9B | 4HZFA | 4HZIA |
| 4I0NA | 4I0OA | 4I0WA | 4I0XA | 4I0XB |
| 4I13B | 4I16A | 4I1HA | 4I1KA | 4I1SB |
| 4I2XE | 4I2YA | 4I35A | 4I3FA | 4I3GA |
| 4I3YA | 4I4CA | 4I4OA | 4I4TA | 4I4TE |
| 4I5JA | 4I5LA | 4I5LB | 4I66A | 4I68A |
| 4I6RA | 4I6XA | 4I6YA | 4I71A | 4I77Z |
| 4I79A | 4I7EA | 4I7WA | 4I84A | 4I8HA |
| 4I8IA | 4I8OA | 4I90A | 4IA6A | 4IABA |
| 4IAJA | 4IAUA | 4IAXA | 4IC3A | 4IC9A |
| 4ICIA | 4ICKA | 4ID3A | 4IDHA | 4IDOA |
| 4IE5A | 4IEFA | 4IEJA | 4IEUA | 4IFLX |
| 4IG1A | 4IGIA | 4IGTA | 4IGVA | 4IHQA |
| 4IHZA | 4IIKA | 4IILA | 4IIYA | 4IJAA |
| 4IJNA | 4IKDA | 4IKNA | 4IKVA | 4IL6A |
| 4IL6B | 4IL6C | 4IL6D | 4IL6E | 4IL6H |
| 4IL6O | 4IL6X | 4IL6Z | 4IL7A | 4ILGC |
| 4IM7A | 4IMQA | 4IN0A | 4INAA | 4INDA |
| 4INKA | 4INWA | 4IO1A | 4IO2A | 4IOOA |
| 4IOYX | 4IPCA | 4IPIA | 4IQMA | 4IQNA |
| 4IQUA | 4IRGA | 4ISVA | 4ISVB | 4IT4A |
| 4ITBA | 4ITRA | 4IU3B | 4IUJA | 4IUMA |
| 4IUPA | 4IUSA | 4IUWA | 4IV5A | 4IVKA |
| 4IVNA | 4IWSA | 4IX3A | 4IYJA | 4IZ7B |
| 4IZBA | 4IZXA | 4J27A | 4J2CA | 4J32A |
| 4J32B | 4J3HA | 4J42A | 4J4HA | 4J4RA |
| 4J4ZA | 4J5RA | 4J6OA | 4J6VA | 4J73A |
| 4J7DA | 4J7HA | 4J7NA | 4J7OA | 4J8CA |
| 4J8SA | 4J9YB | 4JB1A | 4JB7A | 4JBUA |
| 4JCCA | 4JCGA | 4JD0A | 4JDEB | 4JDNA |
| 4JDUA | 4JE3A | 4JE3B | 4JEAA | 4JEMA |
| 4JERA | 4JF3A | 4JF5A | 4JG1H | 4JG1L |
| 4JG2A | 4JG9A | 4JGIA | 4JGLA | 4JHCA |
| 4JHTA | 4JIFA | 4JIUA | 4JIVA | 4JIVD |
| 4JJ0A | 4JJ7A | 4JJAA | 4JJPA | 4JK8A |
| 4JM1A | 4JMDA | 4JMUA | 4JN3A | 4JN7A |
| 4JNDA | 4JO1L | 4JO4L | 4JODA | 4JOQA |
| 4JP6A | 4JPNA | 4JQFA | 4JQPA | 4JQRA |
| 4JQSA | 4JQTA | 4JQUB | 4JR6A | 4JRFA |
| 4JS1A | 4JT4A | 4JTIA | 4JTMA | 4JUIA |
| 4JV8B | 4JVUA | 4JW0A | 4JW2A | 4JWOA |
| 4JXMA | 4JXRA | 4JXUA | 4JZPA | 4JZYA |
| 4JZZA | 4K00A | 4K08A | 4K2JA | 4K2PA |
| 4K30A | 4K35A | 4K37A | 4K3GA | 4K4KA |
| 4K60A | 4K6NA | 4K73A | 4K7BA | 4K84A |
| 4K8WA | 4K90A | 4K90B | 4K92A | 4K94C |
| 4KA7A | 4KAVA | 4KBBC | 4KC5A | 4KDSA |
| 4KDWA | 4KEFA | 4KEMA | 4KF9A | 4KFUA |
| 4KG3A | 4KGDA | 4KH8A | 4KH9A | 4KHAA |
| 4KI0F | 4KI0G | 4KJMA | 4KK7A | 4KKIA |
| 4KKRA | 4KL5A | 4KM6A | 4KMDA | 4KMRA |
| 4KNCA | 4KNUA | 4KO8A | 4KOPA | 4KP3C |
| 4KP3E | 4KQ7A | 4KQ9A | 4KQCA | 4KQPA |
| 4KQWA | 4KRGA | 4KRRA | 4KRXA | 4KSNA |
| 4KT3A | 4KT3B | 4KT6A | 4KT6B | 4KTBA |
| 4KTWA | 4KTYA | 4KUIA | 4KUNA | 4KV7A |
| 4KVSA | 4KVXA | 4KWDA | 4KWYA | 4KXVA |
| 4KY9A | 4KYQA | 4KYSA | 4KZPA | 4L0JA |
| 4L0NA | 4L1UA | 4L2HA | 4L3KA | 4L3NA |
| 4L3RA | 4L3TA | 4L3UA | 4L4EA | 4L51A |
| 4L5EA | 4L63A | 4L6DA | 4L7IA | 4L7XA |
| 4L8AA | 4L8NA | 4L8UA | 4L9AA | 4L9NA |
| 4L9ZA | 4LANA | 4LASH | 4LASL | 4LB0A |
| 4LB8A | 4LBAA | 4LD1A | 4LD3B | 4LDAA |
| 4LDCA | 4LDMA | 4LE3A | 4LERA | 4LEVA |
| 4LF0A | 4LFHD | 4LFHG | 4LG3A | 4LG8A |
| 4LGCA | 4LGYA | 4LHDA | 4LHSA | 4LIMA |
| 4LIRA | 4LIXA | 4LJ1A | 4LJHA | 4LJOA |
| 4LLEA | 4LLOA | 4LMIA | 4LO6A | 4LO6B |
| 4LOSA | 4LP4A | 4LP8A | 4LPQA | 4LPSA |
| 4LQ6A | 4LQ8A | 4LQBA | 4LQKA | 4LQXA |
| 4LQZA | 4LR4A | 4LRTA | 4LRTB | 4LRUA |
| 4LSMA | 4LUQA | 4LUQC | 4LV5A | 4LV5B |
| 4LVFA | 4LW2A | 4LWRA | 4LX2A | 4LXQA |
| 4LY1A | 4LZKA | 4M0JA | 4M0MA | 4M0NA |
| 4M1HA | 4M1UA | 4M43L | 4M4PA | 4M51A |
| 4M5RA | 4M7RA | 4M7TA | 4M83A | 4M88A |
| 4M8KA | 4MAAA | 4MAKA | 4MAQA | 4MAXA |
| 4MB7A | 4MBOA | 4MBYA | 4MCJA | 4MCOA |
| 4MCXB | 4MD5B | 4MDWA | 4MDYA | 4MF5A |
| 4MHCA | 4MIJA | 4MJ2A | 4MJDA | 4MJFA |
| 4MJRA | 4ML1A | 4MLLA | 4MLWA | 4MLZA |
| 4MMSA | 4MNCA | 4MNUA | 4MOZA | 4MPTA |
| 4MQDA | 4MQLA | 4MS4B | 4MT2A | 4MTLA |
| 4MU9A | 4MUBA | 4MUDA | 4MUQA | 4MV2A |
| 4MVEA | 4MWAA | 4MWIA | 4MXNA | 4MXTA |
| 4MZ2A | 4MZ6E | 4MZ7A | 4MZAA | 4MZCA |
| 4MZJA | 4MZYA | 4N01A | 4N03A | 4N0LA |
| 4N0PA | 4N0RA | 4N0VA | 4N2XA | 4N30A |
| 4N3OA | 4N3PA | 4N4JA | 4N4UA | 4N5BA |
| 4N5XA | 4N6DA | 4N6HA | 4N6KA | 4N6PA |
| 4N6WA | 4N70A | 4N7QA | 4N7WA | 4N8GA |
| 4NARA | 4NAWA | 4NAWB | 4NBIA | 4NBXA |
| 4NF0A | 4NFUA | 4NHBA | 4NHEA | 4NI3A |
| 4NI6A | 4NKPA | 4NLMA | 4NMWA | 4NMXA |
| 4NMYA | 4NOCA | 4NOFA | 4NOGA | 4NOHA |
| 4NOIA | 4NPBA | 4NQRA | 4NSMA | 4NV1A |
| 4NV4A | 4NXVA | 4OVJA | 4OVKA | 4PROC |
| 4SGBI | 4TSVA | 7A3HA | 7ODCA | 8ABPA |
| 8ACNA |  |  |  |  |

**S2 The PDB IDs of DB101**

| 4QGKA | 4QC6A | 4QYTA | 3WYNA | 4PWZA |
| --- | --- | --- | --- | --- |
| 4Q82A | 4Q2WA | 4Q3HA | 4OH0A | 4QDJA |
| 4QAGA | 4Q1EA | 3WVSA | 4OAEA | 4Q0KA |
| 4Q7EA | 4QOSA | 4PV2A | 4PV4A | 4OA3A |
| 4OH7A | 4OFIA | 4Q6OA | 4PWTA | 4R9FA |
| 4Q6BA | 4Q6WA | 4QA9A | 4QYXA | 4QMEA |
| 4RG3A | 4PTXA | 4REUA | 4OD7A | 4PTBA |
| 4ONWA | 4OIHA | 4R9NA | 4R9XA | 4R12A |
| 3WT4A | 4PZKA | 4Q0NA | 4OOEA | 4RASA |
| 4QBUA | 4Q29A | 4RCOA | 4RFBA | 4PZLA |
| 4QSPA | 4OFXA | 4OO0A | 4OANA | 4OLSA |
| 4QMHA | 4OFKA | 4QPLA | 4PSYA | 4QC2A |
| 4Q6JA | 3WRBA | 4OCVA | 4Q88A | 4QWOA |
| 4QKDA | 4PUIA | 4Q0IA | 4R81A | 4RO6A |
| 4QHEA | 4PQQA | 4RAWA | 4RE5A | 4RW0A |
| 4RIFA | 4S2VA | 4RI6A | 3X1BA | 4QY2A |
| 4OICA | 4OHNA | 4QVHA | 4QUNA | 4QT7A |
| 4RV8A | 3WY9A | 4ROTA | 4ONIA | 4RKNA |
| 4OBSA | 4OICB | 4S15A | 4QS8A | 4RWHA |
| 4QIBA | 4R4XA | 4OF4A | 4OJAA | 4OJFL |
| 4OECA |  |  |  |  |
